# Is the Phenylalanine-Restricted Diet a Risk Factor for Overweight or Obesity in Patients with Phenylketonuria (PKU)? A Systematic Review and Meta-Analysis

**DOI:** 10.3390/nu13103443

**Published:** 2021-09-28

**Authors:** Catarina Rodrigues, Alex Pinto, Ana Faria, Diana Teixeira, Annemiek M. J. van Wegberg, Kirsten Ahring, François Feillet, Conceição Calhau, Anita MacDonald, André Moreira-Rosário, Júlio César Rocha

**Affiliations:** 1 Nutrition and Metabolism, NOVA Medical School, Faculdade de Ciências Médicas, Universidade NOVA de Lisboa, 1169-056 Lisboa, Portugal; catarina.rodrigues@nms.unl.pt (C.R.); ana.faria@nms.unl.pt (A.F.); diana.teixeira@nms.unl.pt (D.T.); ccalhau@nms.unl.pt (C.C.); 2 Comprehensive Health Research Centre, Universidade NOVA de Lisboa, 1169-056 Lisboa, Portugal; 3 Dietetic Department, Birmingham Children’s Hospital, Steelhouse Lane, Birmingham B4 6NH, UK; alex.pinto@nhs.net (A.P.); anita.macdonald@nhs.net (A.M.); 4 CINTESIS—Center for Health Technology and Services Research, NOVA Medical School, Campo dos Mártires da Pátria 130, 1169-056 Lisboa, Portugal; 5 Division of Metabolic Diseases, Beatrix Children’s Hospital, University Medical Centre Groningen, University of Groningen, Hanzeplein 1, 9700 RB Groningen, The Netherlands; a.m.j.van.wegberg@umcg.nl; 6 Department of PKU, Copenhagen University Hospital, DK-2600 Glostrup, Denmark; kirsten.ahring@regionh.dk; 7 Department of Paediatrics, Reference Center for Inborn Errors of Metabolism, Hôpital d’Enfants Brabois, CHU Nancy, 54500 Vandoeuvre les Nancy, France; f.feillet@chru-nancy.fr; 8 Reference Centre of Inherited Metabolic Diseases, Centro Hospitalar Universitário de Lisboa Central, 1169-045 Lisboa, Portugal

**Keywords:** body mass index, obesity, overweight, phenylalanine restriction, phenylalanine-restricted diet, phenylketonuria

## Abstract

Although there is a general assumption that a phenylalanine (Phe)-restricted diet promotes overweight in patients with phenylketonuria (PKU), it is unclear if this presumption is supported by scientific evidence. This systematic review aimed to determine if patients with PKU are at a higher risk of overweight compared to healthy individuals. A literature search was carried out on PubMed, Cochrane Library, and Embase databases. Risk of bias of individual studies was assessed using the Quality Assessment Tool for Observational Cohort and Cross-Sectional Studies, and the quality of the evidence for each outcome was assessed using the NutriGrade scoring system. From 829 articles identified, 15 were included in the systematic review and 12 in the meta-analysis. Body mass index (BMI) was similar between patients with PKU and healthy controls, providing no evidence to support the idea that a Phe-restricted diet is a risk factor for the development of overweight. However, a subgroup of patients with classical PKU had a significantly higher BMI than healthy controls. Given the increasing prevalence of overweight in the general population, patients with PKU require lifelong follow-up, receiving personalised nutritional counselling, with methodical nutritional status monitoring from a multidisciplinary team in inherited metabolic disorders.

## 1. Introduction

In phenylketonuria (PKU), the prevalence and patient susceptibility to overweight and obesity has been widely discussed. Several retrospective studies have reported a higher body mass index (BMI) and a higher prevalence of overweight in patients with PKU compared to the normal population [1,2,3,4], especially in females [1,5,6,7,8,9]. Generally, the prevalence of overweight worldwide has almost tripled since 1975 [10]. This multifactorial comorbidity is mainly associated with poor dietary habits and lack of physical activity, but other factors, such as social economic status and family history, may also influence outcome [11].

The World Health Organisation (WHO) defines overweight and obesity as abnormal or excessive fat accumulation. This has numerous negative health consequences including cardiovascular diseases, non-insulin-dependent diabetes mellitus, musculoskeletal disorders, pulmonary diseases, and cancer [12,13,14].

PKU is a rare autosomal recessive inborn error of phenylalanine (Phe) metabolism, and if untreated, can cause severe and irreversible neurological damage [15]. The main treatment is a Phe-restricted diet, composed of three parts: (1) strict control of natural protein intake according to individual Phe tolerance, (2) administration of a synthetic protein derived from Phe-free amino acids (L-AAs) or low-Phe glycomacropeptide supplemented with amino acids (GMP-AA), and (3) and low-Phe foods including the use of special low-protein foods (SLPFs). The primary aim is to prevent neurological sequelae by maintaining blood Phe levels within a therapeutic target range [14], whilst maintaining nutritional requirements to achieve normal growth and body composition.

Adequate dietary energy is essential to maintain blood Phe stability, particularly in patients with classical PKU, by promoting anabolism and counteracting catabolism, which increases blood Phe levels [15]. Energy is obtained from fruits and some vegetables, sugars, fats, and oils, as well as SLPFs such as bread, pasta, rice, cereals, and milk replacements, aiming to replace regular foods. Pena et al. [16] analysed the food labels of several SLPFs and found that, when compared to their regular foods, 75% had a higher energy content, 58% a higher fat content, and 92% a higher carbohydrate (CHO) content. Moreover, the quality of fat and fibre differs from regular foods [17]. Their consumption without moderation may lead to excessive energy intake, with a low supply of micronutrients, although these are usually supplied by protein substitutes (PS) [18,19]. Overall, a Phe-restricted diet is characterised by higher CHO intake compared with the general population [19,20].

Due to concerns over increasing obesity in PKU, industry has reformulated many of their PS, adding less CHO to their products [21]. Furthermore, a higher prevalence of overweight in patients with PKU is used to support the need for alternative treatments, even though a systematic analysis of published data is not available to verify this claim. In addition, some studies have found no differences in BMI and prevalence of overweight and obesity between patients with PKU and healthy individuals [22,23,24,25,26].

This lack of consensus highlights the need to assess the quality of evidence that reports the prevalence of overweight and obesity in PKU. This systematic review aims to (1) determine if patients with PKU are at a higher risk of overweight compared to healthy individuals, and to (2) understand the association between early exposure to Phe restriction and overweight in patients with PKU.

## 2. Materials and Methods

### 2.1. Protocol and Registration

This systematic review with meta-analysis was developed according to preferred reporting items for systematic reviews and meta-analyses (PRISMA) statement [27] and the Cochrane Handbook for Systematic Reviews of Interventions [28] guidelines. The protocol was registered (CRD42020214436) in the International Prospective Register of Systematic Reviews (PROSPERO).

### 2.2. Selection Criteria

Inclusion and exclusion criteria were defined according to the PECO (Population, Exposure, Comparator, Outcome) strategy. Inclusion criteria: (1) patients with PKU (Population) on a Phe-restricted diet (Exposure) and followed up at a PKU centre; (2) studies included healthy controls (Comparator); (3) reported anthropometric measures or prevalence of overweight (Outcome); (4) published as a full paper; and (5) included only randomised controlled trials (RCTs), non-randomised controlled trials (non-RCTs), or observational (case–control, cohort, and cross-sectional) studies.

Non-human studies, review articles, systematic reviews, meta-analysis, letters, conference abstracts, case reports, case series, position papers, and authors’ replies were excluded. Only studies published in English were included.

### 2.3. Search Strategy

A literature search was carried out on PubMed, The Cochrane Library, and Embase databases on the 16 January 2020. Both medical subject headings (MeSH or Emtree) and text words related to overweight, obesity, and PKU were used. The PubMed search strategy was converted to search in other databases as described in detail in the Supplementary Materials, Section A.

### 2.4. Study Selection

All articles identified in the search were included in the screening process and duplicates excluded. Two independent reviewers (A.M. and J.C.R.) screened the titles and abstracts of the articles for relevance, and full-text articles were reviewed when title and abstract did not provide enough information. Once potentially relevant studies were identified, full-text articles were then assessed for eligibility according to previously established criteria. The reference lists of the included articles were screened to ensure that no relevant studies were missed.

### 2.5. Data Extraction

Data items were extracted by two authors (C.R. and A.P.) using a standard data extraction form. For each study, first author, year of publication, country of origin, study design, sample characteristics, methods, and outcomes were extracted. In cases where information was missing or incomplete, the correspondence authors were contacted requesting further information.

### 2.6. Assessment of Risk of Bias in Individual Studies

Risk of bias of individual studies was assessed by two independent reviewers (C.R. and A.P.) using the National Institutes of Health (NIH) Quality Assessment Tool for Observational Cohort and Cross-Sectional Studies [29]. The following domains were assessed: (1) research question; (2) study population; (3) eligibility criteria; (4) justification of the sample size; (5) exposure measures and assessment; (6) time frame between exposure and outcome assessment; (7) outcome measures; (8) blinding of outcome assessors; (9) follow-up rate; and (10) adjustment of confounders. Reviewers were blinded to each other’s assessment, and disagreements were solved by reaching consensus.

### 2.7. Quantitative Synthesis

Standardised mean difference (SMD) was used as an effect measure for the continuous variable ‘BMI’. Odds ratio (OR) was used as an effect measure for the dichotomous variable ‘prevalence of overweight’. The SMD and OR were converted to a common metric and then combined across studies. A sensitivity analysis was performed to compare the meta-analysis results with and without the converted study [30]. Effect measures were reported along with the 95% confidence interval (CI).

The Cochran’s Q (significance level of 0.1) and I^2^ tests were used to assess heterogeneity. According to the Cochrane guidelines [28], the I^2^ values were interpreted as follows: 0% to 40% might not be important; 30% to 60% may represent moderate heterogeneity; 50% to 90% may represent substantial heterogeneity; 75% to 100% represent considerable heterogeneity.

Mean BMI from Evans et al. [31] was calculated with values from the last evaluation (longest time-point of exposure). In the studies from Evans et al. [25] and Huemer et al. [26], only the mean BMI from the first evaluation (baseline) could be included. In the study from Schulpis et al. [32], consisting of patients both adhering to their diet and on a ‘relaxed diet’, only the BMI of the patients adhering to the diet was included in the meta-analysis.

Pooled estimates were computed and weighted using generic inverse-variance and random-effect modelling. A *p*-value < 0.05 was considered as statistically significant. Statistical analysis was performed using Review Manager (RevMan), version 5.4, The Cochrane Collaboration, 2020.

### 2.8. Grading the Evidence

Funnel plots were used to assess evidence of publication bias. Quality assessment of the evidence for each outcome was performed by two independent authors (C.R. and A.P.) using the NutriGrade scoring system [33]. The meta-analysis was scored with a maximum of 10 points, according to (1) risk of bias, (2) precision, (3) heterogeneity, (4) directness, (5) publication bias, (6) funding bias, (7) effect-size, and (8) dose–response. On the basis of the final score, we classified the quality of the evidence as high, moderate, low, or very low.

## 3. Results

### 3.1. Study Selection

**Figure 1:**
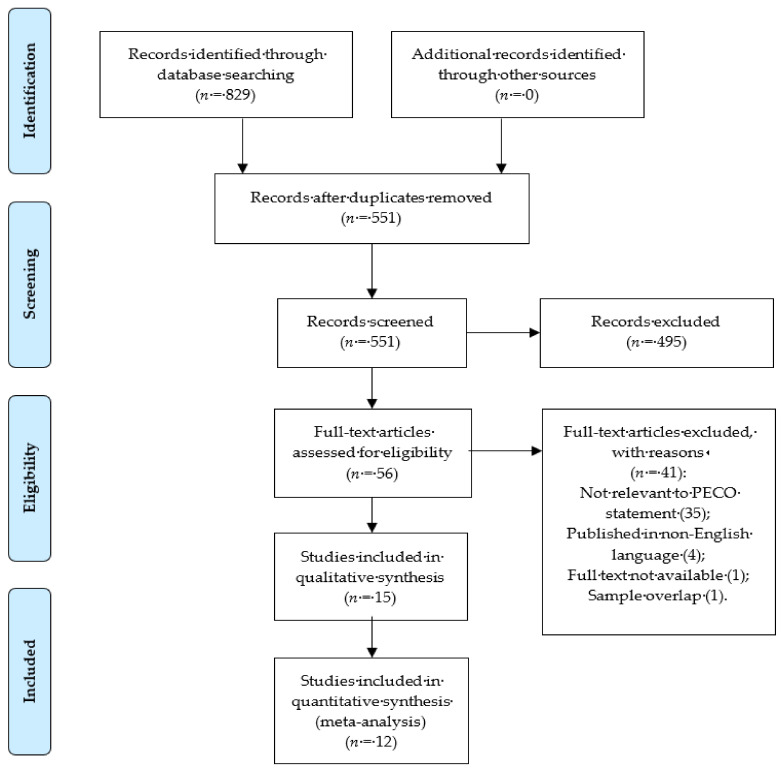
PRISMA study flow diagram describing the process of study selection. Abbreviation: PECO: Population, Exposure, Comparator, Outcome.

A total of 829 articles were identified through database search (Figure 1). Titles and abstracts of 551 articles were screened for relevance, after removing duplicates. Once potentially relevant studies were identified, a total of 56 full-text articles were assessed for eligibility. Studies not fulfilling these criteria were excluded from the analysis (*n* = 41) (Supplementary Materials, Section B). Two studies by Rocha et al. [22,34] included two overlapping patient cohorts. To avoid duplicate publication bias, we included the study with more complete information [34]. From the included studies, only 12 provided data on BMI or the prevalence of overweight, qualifying them for quantitative analysis [7,18,25,26,30,31,32,34,35,36,37,38].

### 3.2. Study Characteristics

A summary of the main characteristics of included studies is given in Table 1. All studies were observational: 11 cross-sectional studies [7,18,30,32,34,35,36,37,38,39,40], 2 cross-sectional with nested longitudinal cohort studies [26,41], and 2 prospective studies [25,31]. Nine studies were conducted in Europe [7,26,30,31,32,34,35,36,37], three in Australia [25,39,41], two in Brazil [38,40], and one in the USA [18]. Studies were published between 1995 and 2020. In prospective studies, duration of follow-up ranged from 1 to 2 years. The total sample size of the 15 studies was 640 patients with PKU, and 503 were included in the meta-analysis (12 studies). All studies included patients with PKU from both genders (301 females and 299 males). Fisberg et al. [40] did not specify children’s gender. The age range of the participants ranged from 2 months to 52 years. Most studies included children and adolescents, four included children, adolescents, and adults [30,34,37,38], and Azabdaftari et al. [36] included adults only.

The methods used to assess dietary intake varied between the included studies and are given in Table 2. No valid and reliable methods to assess exposure were used in five studies [7,35,37,38,39].

Patients with PKU were compared to 593 healthy controls, 455 of which were included in the meta-analysis. Healthy controls were from both genders, and the age range varied from 1 month to 50 years. The majority were matched for age and gender, and some studies included family relatives, friends, or healthy individuals with similar characteristics in the PKU group.

Most studies examined the association between a Phe-restricted diet and BMI [7,18,25,26,31,32,34,35,36,37,38]. Six studies examined the association between a Phe-restricted diet and overweight prevalence [18,30,31,34,37,38]. Eleven studies examined the association of different or additional parameters, such as weight-for-height and weight z-scores and body fat percentage [7,18,25,26,31,34,35,38,39,40,41].

From 15 studies included in the qualitative synthesis, 12 did not find significant differences in BMI and overweight prevalence between patients with PKU on a Phe-restricted diet, compared with healthy controls [7,18,25,26,31,32,34,37,38,39,40,41] (Table 1). Only 3 of 15 studies found a significantly higher BMI or higher prevalence of overweight in patients with PKU than controls [30,35,36].

**Table 2:** Exposure assessment method and nutritional intake of participants in the included studies.

Reference	Exposure Assessment Method	Natural Protein (g/kg/day)	PE from PS Supplements (g/kg/day)	Carbohydrate (%)	Lipids (%)	Energy (kcal)	BH4 Treatment	Additional Information
Allen et al., 1995 [39]	NA	NA	NA	NA	NA	NA	No	-
Allen et al., 1996 [41]	4 day dietary records	2.1		NA	NA	1.6xBMR	No	
Fisberg et al., 1999 [40]	3 day dietary records	<7 y: 105.0% RDA ^1^ ≥7 y: 109.4% RDA ^1^		NA	NA	<7 y: 62.6% RDA ≥7 y: 60.5% RDA	No	-
Schulpis et al., 2000 [32]	1 week dietary record + 24 h dietary recall	strict diet: 7.5 ± 5.6 g/day; ‘loose’ diet: 15.8 ± 5.5 g/day	strict diet: 60.6 ± 7 g/day; ‘loose’ diet: 55.1 ± 14 g/day	strict diet: 49 ‘relaxed’ diet: 43	strict diet: 21 ‘loose’ diet: 38	strict diet: 2114 ± 463 ‘loose’ diet: 2080 ± 487	No	-
Huemer et al., 2007 [26]	3 day dietary records	0.3	0.9	NA	NA	NA	No	-
Albersen et al., 2010 [7]	NA	1.3–1.5 times above RDA ^1^		NA	NA	-	No	-
Rocha et al., 2012 [34]	Food history from the nutrition appointment	HPA: 1.16 ± 0.53 MPKU: 0.59 ± 0.33 CPKU: 0.59 ± 0.36	HPA: 1.13 ± 0.41 MPKU: 1.38 ± 0.43 CPKU: 1.25 ± 0.53	HPA: 58 ± 5 MPKU: 60 ± 4 CPKU: 58 ± 6	HPA: 30 ± 5 MPKU: 25 ± 4 CPKU: 26 ± 4	HPA: 2260 ± 332 MPKU: 2351 ± 391 CPKU: 2451 ± 316	No	-
Doulgeraki et al., 2014 [35]	NA	NA	NA	NA	NA	No	No	HPA on free diet
Mazzola et al., 2016 [38]	NA	NA	NA	NA	NA	NA	No	-
Evans et al., 2017 [25]	Food diary	0.50 ± 0.18	1.54 ± 0.50	NA	NA	1665 ± 546	Yes (5 patients)	-
Hermida-Ameijeiras et al., 2017 [37]	NA	NA	NA	NA	NA	NA	Yes (7 patients)	-
Couce et al., 2018 [30]	3 day dietary records	1.3–1.5 times above RDA ^1^		CPKU: 57.0 ± 8.6 MPKU: 53.5 ± 9.8	NA	NA	Yes (10 patients)	HPA on free diet
Evans et al., 2019 [31]	1 day dietary record	0.43 ± 0.26	2.75 ± 0.39 ^2^	60 ^2^	25 ^2^	1320 ^2^	No	-
Azabdaftari et al., 2019 [36]	3 day dietary records	0.19 ± 0.13	0.73 ± 0.21	NA	NA	NA	No	-
Sailer et al., 2020 [18]	24 h dietary recall	0.39 ± 0.31	1.10 ± 0.72	67 ± 9	24 ± 8	2356 ± 620	Yes (4 patients)	-

Abbreviations: BH4: sapropterin; BMR: basal metabolic rate; CPKU: classical PKU; G: grams; HPA: hyperphenylalaninaemia; Kcal: kilocalorie; Kg: kilograms; MPKU: mild–moderate PKU; NA: not available; PE: protein equivalent; PS: protein substitute; RDA: recommended dietary allowances; y: years. ^1^ Total protein (g/kg/day); ^2^ at 24 months of age. Examining PKU phenotype, five studies included only patients with classical PKU [7,18,26,32,41], seven mixed phenotypes [30,31,34,35,36,37,38], and three did not specify [25,39,40].

### 3.3. NutriGrade Assessment

On the basis of the NutriGrade assessment (Supplementary Materials, Section C—Table S5), we found that the quality of the evidence for the meta-analysis using BMI was low, with meta-evidence limited and uncertain. The quality of the evidence for the meta-analysis using body fat percentage was very low, with meta-evidence very limited and uncertain.

### 3.4. Risk of Bias Assessment

**Figure 2:**
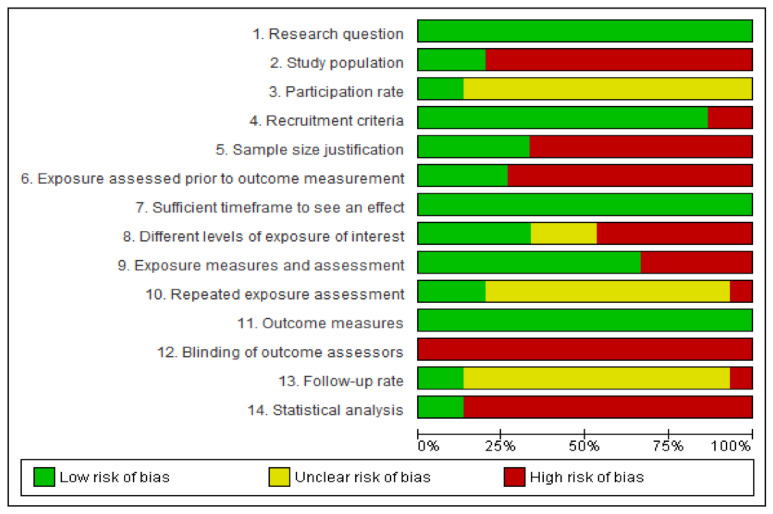
Risk of bias: judgements about each risk of bias item presented as percentages across all included studies.

Using the NIH Quality Assessment Tool for Observational Cohort and Cross-Sectional Studies, we found that 4 studies were assessed as fair with moderate risk of bias [26,30,31,34], and 11 as poor with high risk of bias [7,18,25,32,35,36,37,38,39,40,41]. Figure 2 presents the percentages of compliance for each tool item across all included studies. The risk of bias summary with review authors’ judgments about each item for all included studies can be found in the Supplementary Materials, Section C—Figure S1.

Visual inspection of the funnel plot did not indicate substantial asymmetry (Supplementary Materials, Section C—Figure S7).

### 3.5. Synthesis of Results

#### 3.5.1. Patients with PKU vs. Healthy Controls

**Figure 3:**
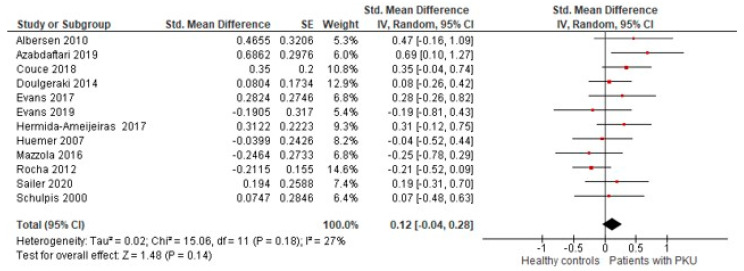
Forest plot comparing the BMI between patients with PKU and healthy controls. Abbreviations: BMI: body mass index; CI: confidence interval; df: degrees of freedom; IV: inverse variance; PKU: phenylketonuria; SE: standard error; Std: standardised. Moderate risk of bias: Couce 2018, Evans 2019, Huemer 2007, and Rocha 2012. High risk of bias: Albersen 2010, Azabdaftari 2019, Doulgeraki 2014, Evans 2017, Hermida-Ameijeiras 2017, Mazzola 2016, Sailer 2020, and Schulpis 2000. Time of diagnosis: Couce 2018 included 70 early and 13 late diagnosed patients, Hermida-Ameijeiras 2017 included both early and late diagnosed patients, Mazzola 2016 included 11 early and 16 late diagnosed patients, and Schulpis 2000 did not provide information on the time of diagnosis. Metabolic control: Azabdaftari 2019 included only one patient with good metabolic control (Phe blood levels < 600 μmol/L). BH4 treatment: Couce 2018 included 10 (12%) patients taking BH4, Evans 2017 included 5 (14%), Hermida-Ameijeiras 2017 included 7 (17%), and Sailer 2020 included 4 (13%).

In the 12 studies included in the meta-analysis, there were no differences for BMI of patients with PKU compared with healthy controls (SMD = 0.12 [−0.04, 0.28], *p* = 0.14; I^2^ = 27%, *p* = 0.18; Figure 3).

#### 3.5.2. Moderate vs. Poor Risk of Bias Studies

A subgroup analysis was conducted according to the risk of bias for each study (Supplementary Materials, Section C—Figure S2). Studies assessed as fair with moderate risk of bias [26,30,31,34] found no difference in BMI between patients and healthy controls (SMD = −0.02 [−0.30, 0.27], *p* = 0.91; I^2^ = 43%, *p* = 0.16). Studies assessed as poor with high risk of bias [7,18,25,32,35,36,37,38] found a significantly higher BMI in patients with PKU compared to healthy controls (SMD = 0.20 [0.03, 0.37], *p* = 0.02; I^2^ = 1%, *p* = 0.42).

#### 3.5.3. Time of Diagnosis

Three studies included late diagnosed patients in their samples [30,37,38], and Schulpis et al. [32] did not provide information on diagnostic age. Thus, a subgroup analysis was conducted according to diagnostic age (Supplementary Materials, Section C—Figure S3). The subgroup of studies including only early diagnosed patients found no differences in BMI between patients and healthy controls (SMD = 0.11 [−0.10, 0.31], *p* = 0.32; I^2^ = 35%, *p* = 0.15). Moreover, the subgroup of studies including both early and late diagnosed patients found no differences between patients with PKU and healthy controls (SMD = 0.18 [−0.17, 0.52], *p* = 0.31; I^2^ = 43%, *p* = 0.18). There were no statistical differences between the two subgroups (*p* = 0.73).

#### 3.5.4. Age

The studies included in the meta-analysis covered a wide patient age. We performed a subgroup analysis (Supplementary Materials, Section C—Figure S4) comparing studies including children and adolescents only [7,18,25,26,31,32,35], adults only [36], and all age groups (children, adolescents, and adults) [30,34,37,38]. We found no differences between the three subgroups (*p* = 0.15), and a higher heterogeneity in the subgroup of studies that included all age groups (I^2^ = 61%). The subgroup that included adults only had one study [36] that identified adult patients with PKU, having a significantly higher BMI when compared to healthy adults.

#### 3.5.5. Sapropterin (BH4) Treatment

Four studies included patients prescribed BH4 in their patient cohort [18,25,30,37]. To understand if there was any difference between studies that included patients taking BH4 (mixed sample) and studies that included only patients on a Phe-restricted diet, we performed a subgroup analysis (Supplementary Materials, Section C—Figure S5).

Studies that included some patients with PKU treated with diet and BH4 [18,25,30,37] found a significantly higher BMI in the overall group than in healthy controls (SMD = 0.30 [0.07, 0.52], *p* = 0.01; I^2^ = 0%, *p* = 0.97). Studies that included only patients on a Phe-restricted diet [7,26,31,32,34,35,36,38] found no differences between the PKU group and healthy controls (SMD = 0.04 [−0.17, 0.24], *p* = 0.74; I^2^ = 35%, *p* = 0.15).

#### 3.5.6. Phenotype

Four studies in the meta-analysis included only patients with classical PKU [7,18,26,32]. The remaining studies included patients with different phenotypes and reported their BMI together; therefore, it was not possible to analyse any association between different phenotypes and BMI from these studies [30,31,34,35,36,37,38]. To understand if there were any differences between studies including only patients with classical PKU and studies that included patients with different phenotypes, we performed a subgroup analysis (Supplementary Materials, Section C—Figure S6). In both subgroups, there were no differences between patients with PKU and controls.

#### 3.5.7. Patients with Classical PKU vs. Healthy Controls

**Figure 4:**
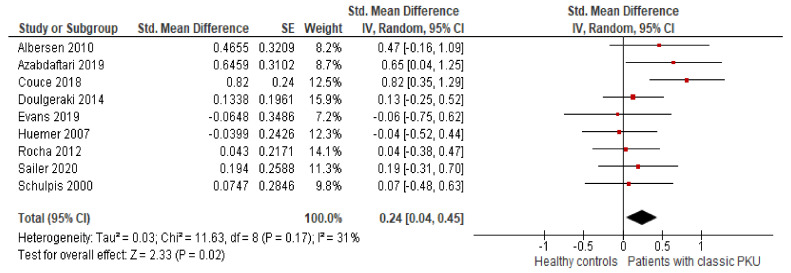
Forest plot comparing the BMI between patients with classical PKU and healthy controls. Abbreviations: BMI: body mass index; CI: confidence interval; df: degrees of freedom; IV: inverse variance; PKU: phenylketonuria; SE: standard error; Std: standardised. Moderate risk of bias: Couce 2018, Evans 2019, Huemer 2007, and Rocha 2012. High risk of bias: Albersen 2010, Azabdaftari 2019, Doulgeraki 2014, Sailer 2020, and Schulpis 2000. Time of diagnosis: Couce 2018 included 70 early- and 13 late-diagnosed patients, and Schulpis 2000 did not provide information on the time of diagnosis. Metabolic control: Azabdaftari 2019 included only one patient with good metabolic control (Phe blood levels < 600 μmol/L). BH4 treatment: Couce 2018 included 1 (3%) patient taking BH4, and Sailer 2020 included 4 (13%) patients.

Several authors of the included studies provided individual participant data, including disease severity [7,18,31,34,35,36]. On the basis of this additional data, we conducted a meta-analysis comparing patients with classical PKU only with healthy controls (Figure 4) [7,18,26,32]. In the remaining studies, we calculated the mean BMI of patients with classical PKU [30,31,34,35,36] and excluded data from patients with other phenotypes. Individual participant data was unavailable from two studies (Hermida-Ameijeiras et al. [37] and Mazzola et al. [38]), and Evans et al. [25] did not include information on the patient phenotype. Therefore, these three studies were excluded from this meta-analysis.

We found that patients with classical PKU had a significantly higher BMI than healthy controls (SMD = 0.24 [0.04, 0.45], *p* = 0.02; I^2^ = 31%, *p* = 0.17).

To reject the hypothesis that this result was due to the removal of the three studies, whose individual participant data is unknown, we performed the first meta-analysis (Figure 3) without them. Removing these three studies did not affect the overall result, compared with the 12 included studies (SMD = 0.12 [−0.07, 0.31], *p* = 0.22; I^2^ = 34%, *p* = 0.15).

#### 3.5.8. Sex

Only six studies provided adequate information to establish a comparison on sex, which limits the subsequent interpretation of its effect on overweight. However, when comparing females with PKU and healthy females, all studies found a trend towards a higher BMI in females with PKU (Supplementary Materials, Section C—Table S4).

#### 3.5.9. Metabolic Control

We tried to explore the association between metabolic control and BMI. However, only five studies provided information on metabolic control, and the comparison between patients with poor metabolic control and healthy controls (Supplementary Materials, Section C—Table S4) had substantial heterogeneity (I^2^ = 58%, *p* = 0.05); thus, we were unable to present accurate data on metabolic control.

#### 3.5.10. Body Fat Percentage

The methods used to assess body fat percentage across studies were different. This led to a heterogeneous overall result, rendering it unfeasible to present and compare body fat results (Supplementary Materials, Section C—Table S4).

## 4. Discussion

### 4.1. Summary of Evidence

To the best of our knowledge, this is the first systematic review with meta-analysis evaluating the association between a Phe-restricted diet and overweight and obesity in patients with PKU. We pooled data from 12 observational studies for the meta-analysis and found no differences between patients with PKU and healthy controls for BMI. The pooled data included diverse patient phenotypes with variable Phe-restriction, with dissimilar contributions from the PS and SLPFs to total protein and energy intake [16,42,43]. Our meta-analysis suggests that dietary Phe-restriction alone is not a risk factor for the development of overweight and obesity.

However, patients with classical PKU had a significantly higher BMI than healthy controls. This observation resulted from nine studies, including only patients with classical PKU and studies whose authors provided additional individual participant data, although these results should be considered with caution. One plausible explanation is that more calories may be given to patients with classical PKU in order to prevent catabolism that causes higher blood Phe levels. This may lead to the development of overweight.

Among the studies included in qualitative synthesis, 4 studies had a moderate risk of bias and 11 had a high risk of bias using the NIH Quality Assessment Tool. The subgroup of studies with moderate risk of bias did not find a higher BMI in patients with PKU. In contrast, studies assessed as poor due to their methodological flaws found a significantly higher BMI in patients with PKU compared to healthy controls. Therefore, this work highlights the fragility of the evidence supporting the idea that a Phe-restricted diet promotes overweight and indicates the need for controlled studies with improved methodology and comprehensive data collection.

Three of the seven most common flaws observed in the studies were limited description of the study population using demographics (who), location (where), and time period (when) (question 2 of the NIH tool) [7,18,25,26,31,32,35,36,38,39,40,41]; absence of sample size justification (question 5 of the NIH tool) [18,25,26,30,32,35,37,38,40,41]; and outcome assessors being aware of participants’ exposure status (question 12 of the NIH tool) in all included studies. These flaws were not considered fatal, and studies that failed these criteria could still be classified as fair with moderate risk of bias.

Eleven studies were cross-sectional [7,18,30,32,34,35,36,37,38,39,40], and the exposure was not assessed prior to outcome measurement (question 6 of the NIH tool). For this reason, it is not possible to establish a relation of causality between the exposure to a Phe-restricted diet and overweight.

For the different levels of exposure assessment (question 8 of the NIH tool), from the 10 studies that included patients with different phenotypes, the use of BH4 with a relaxed Phe-restriction or patients who were late diagnosed with PKU, only five studies considered these factors [25,30,32,34,35]. These different levels of exposure to the Phe-restricted diet renders it difficult to analyse the association between the Phe-restricted diet and overweight. For example, we identified three studies that included patients with HPA [30,34,35] and, in two of three of these studies, patients were on an unrestricted diet [30,35]. The fact that most studies included patients with different phenotypes does not allow for conclusions about the association between phenotype and overweight, as verified in the subgroup analysis by phenotypes (Supplementary Materials, Section C—Figure S6).

In addition, between 20 and 50% of patients with PKU are responsive to the synthetic form of the cofactor (BH4), meaning that a less restricted diet is followed. Evidence suggests that 51% of patients on BH4 therapy completely stop PS intake [44]. In our meta-analysis, the studies that included patients taking both BH4 combined with patients on a traditional Phe-restricted diet only found a significantly higher BMI in the overall group of patients with PKU compared to healthy controls. Although this is an interesting finding, it is unknown as to how many of these patients were overweight before BH4 commencement. A study conducted in Spain, including patients from 13 hospitals, found that patients taking BH4 had significantly higher BMI z-scores than patients on a Phe-restricted diet only, with follow up consistently over 2 years [45]. These results highlight the need for a continuous nutritional monitoring and specialised nutritional care, even in patients under pharmacological treatment. This observation warrants further study.

Of the 12 studies included in the meta-analysis, 4 did not assess patients’ dietary intake [7,35,37,38]. In the remaining eight studies, the methods used to assess intake were different, and only four studies [18,31,32,34] provided detailed information on the amount of protein, CHO, fat, and energy patients consumed. This information is central to accurately address our review question and is considered an important omission in studies. Different reimbursement policies in different countries determine access to PS and SLPFs, which ultimately will alter the intake of macronutrients supplied by a Phe-restricted diet [46,47].

We also tried to determine if there was an association between patients’ BMI and metabolic control (which may reflect patients’ exposure to the Phe-restricted diet). However, most of the studies did not report patients’ BMI, nor its comparison with metabolic control. In the literature, some studies have found a positive correlation between mean Phe levels and BMI [3,36,48], and between mean Phe levels and the prevalence of overweight [1,9,34], indicating that good metabolic control is associated with a lower risk of overweight. Conversely, two studies from Spain found a higher prevalence of overweight and BMI in patients with good metabolic control compared to poorly controlled patients [30,49].

Most of the included studies did not adjust for key prognostic variables, such as physical activity, family history, socioeconomic status, parents’ weight, and epigenetics, among other determinant factors that may be associated with overweight.

Finally, none of the included studies considered the regular follow-up of patients by a nutritionist. Nutritionists play a crucial role in monitoring the patient’s weight while ensuring they meet their complex dietary needs [50]. Consequently, we were not only analysing the influence of the Phe-restricted diet alone on overweight, but also on the quality of the follow-up that the patients receive.

### 4.2. Strengths and Limitations of This Study

Several limitations in this systematic review should be acknowledged. First, our systematic review included observational studies only. Observational evidence usually provides lower strength evidence than RCTs, due to confounding variables. Nevertheless, RCTs addressing our question have not been conducted, which is unsurprising, given that PKU is a rare disease and the exposure to an unrestricted Phe-diet is clinical and ethically unacceptable. In addition, there was large heterogeneity in the design of observational studies and in the reporting of results.

The diversity of the study populations also contributes to the heterogeneity of the results. For instance, some studies included patients with different disease severities, with variable degrees of Phe-restriction, being diagnosed early and later on, patients on BH4 treatment, and patients with poor metabolic control. Additionally, patients had a wide age range.

The Phe-restricted diet was not always well defined: not all studies reported patients’ dietary intake, and some studies did not assess it.

In relation to the comparator, we did not define any inclusion criteria for healthy controls. Most of them were matched for age and gender only, and the number of controls included in our work was less than the number of patients with PKU.

Regarding the outcome, one study [30] only presented the prevalence of overweight, which led us to convert the respective OR to a SMD to include it in the meta-analysis. Although BMI is an important predictor of adiposity and is a tool widely used in clinical practice [23], it may not always identify individuals with increased fat mass percentage [51], which underlines the weakness of the BMI as an indicator of adiposity. Measuring body composition appears to be a better approach to identify individuals with increased fat mass percentage, specifically those at a higher risk of metabolic complications, which is crucial to help prevent the development of comorbidities [51]. Increased abdominal obesity is associated with dyslipidaemia, hypertension, insulin resistance, and inflammation.

Finally, most of the included studies had a high risk of bias according to the NIH tool. On the basis of the NutriGrade assessment, we found that the quality of the meta-analysis comparing all patients with PKU to controls was ‘low’, and the quality of the meta-analysis comparing patients with classical PKU to controls was ‘very low’.

In order to strengthen the conclusions of our systematic review with meta-analysis, we used the best methodology, namely, (1) following the PRISMA guidelines and registering on the PROSPERO database—studies that do appear to be of higher quality [27,52]; (2) clear definition of the aim of our work; (3) clear definition of the inclusion and exclusion criteria, according to the PECO strategy; (4) using several databases for the search and searching reference lists of the retrieved studies; (5) describing the study selection process using a flow diagram; (6) providing the list of the excluded studies and the reasons; (7) providing of the characteristics of individual studies; (8) contacting the correspondence authors to request further information; (9) performing meta-analysis and subgroup analysis; and (10) having two independent authors performing study selection, data extraction, and assessment of the risk of bias and the quality of the evidence.

As the study of risk factors is based on comparisons between exposed and unexposed individuals [53], only studies with a control group were included in our systematic review, which is another strength of this meta-analysis. Indeed, several studies that propose that the Phe-restricted diet promotes overweight did not include a control group.

Finally, our systematic review provides a clear overview of the available evidence on the topic overweight and PKU and will be useful in guideline development. It also identifies the main flaws and pitfalls that should be avoided when designing novel studies to address this question in the future.

## 5. Conclusions

We found no differences between patients with PKU and healthy controls in BMI. Thus, there is no evidence to support the concept of Phe-restricted diet as a risk factor for the development of overweight. However, a subgroup of patients with classical PKU had a significantly higher BMI than healthy controls. In addition, studies assessed as poor with high risk of bias and studies that included both diet-treated and BH4-treated patients found a significantly higher BMI in patients with PKU compared to healthy controls.

Given the increasing prevalence of overweight in the general population, patients with PKU should remain in long-term follow-up, receiving personalised nutritional advice with systematic nutritional status monitoring by a multidisciplinary team in inherited metabolic disorders. This is essential to prevent overweight, obesity, and its related comorbidities.

Future studies with improved methodology are needed to properly address this question and to help in guiding the clinical practice of health professionals.

## Figures and Tables

**Table 1:** Characteristics of the studies included in the systematic review.

Reference	Country	Study Design (Duration of Follow-Up)	Sample Size (Phenotype)	Early and Continous Treatment	Age Range (Years)	Gender (F:M)	Annual Phe Levels (μmol/L)	Controls (Type)	Outcomes (Units)	Key Findings	Risk of Bias ^1^
Allen et al., 1995 [39]	Australia	Cross-sectional	30 (NA)	NA	4.6–17.0	15:15	NA	76 family relatives, (age range: 4.3–18.4 y)	Body fat (%) Weight z-score	No significant differences between males with PKU and control subjects for weight scores, body fat, or fat free mass. Females with PKU had lower fat free mass and there was no difference in weight scores and body fat.	High
Allen et al., 1996 [41]	Australia	Cross-sectional with longitudinal cohort (1.1 y)	37 (37 classical)	Yes (NBS)	3.9–11.0 ^2^	16:21	median at the time of the study: 652	27 unaffected siblings (PKU or cystic fibrosis), (age range: 4.0–11.5 y)	Body fat (%) Weight z-score	No significant differences between children with PKU and controls for body fat, lean body mass, or weight. Children with PKU were significantly shorter than controls.	High
Fisberg et al., 1999 [40]	Brazil	Cross-sectional	42 (NA)	NA	1.0–12.0	NA (both genders)	NA	31 with similar characteristics (age range: 1.0–12.0 y)	Weight for height z-score Weight z-score	No significant differences between patients with PKU and controls for weight for height and weight for age z-scores.	High
Schulpis et al., 2000 [32]	Greece	Cross-sectional	49 (49 classical–21 strict diet + 28 ‘relaxed’ diet)	NA	strict diet: 5.2 ± 1.4 ‘relaxed’ diet: 6.0 ± 1.5 (mean + SD)	23:26	mean ± SD: strict diet: 150 ± 40 ‘loose’ diet: 800 ± 40	30 with similar age (mean age + SD: 7.9 ± 1.2 y)	BMI (kg/m^2^)	No significant difference for BMI between patients with PKU adhering to their diet or on a ‘relaxed diet’ and controls. Patients with PKU on a ‘relaxed diet’ had significantly higher leptin concentrations compared to patients with PKU adhering to their diet and controls.	High
Huemer et al., 2007 [26]	Austria	Cross-sectional with longitudi- nal cohort (1 y)	34 (34 classical)	Yes (NBS)	0.2–15.0 ^2^	12:22	mean ± SD at the time of the study: <10 y: 456 ± 432 10–15 y: 534 ± 324 >15 y: 444 ± 228	34 matched for age and gender (mean age difference: 0.5 y)	BMI (kg/m^2^) BMI z-scores Weight z-score	No significant differences for BMI and body fat mass between patients with PKU and controls.	Moderate
Albersen et al., 2010 [7]	The Netherlands	Cross-sectional	20 (20 classical)	Yes (NBS)	6.0–16.0	13:7	mean ± SD: 375 ± 253?(F: 420 ± 303; M: 291 ± 77)	20 matched for age and gender (mean age difference: 0.5 y)	BMI (kg/m^2^) Body fat (%)	No significant differences between children with PKU and controls for body weight and BMI. Body fat % was significantly higher in patients with PKU, especially in girls aged > 11 years.	High
Rocha et al., 2012 [34]	Portugal	Cross-sectional	89 (29 classical, 42 mild 18 HPA)	Yes (NBS)	3.0–30.0	41:48	mean ± SD: 393 ± 245	79 siblings, family or friends (mean age difference: 1.9 y)	BMI (kg/m^2^) Body fat (%) Overweight prevalence (%)	No significant differences between patients with PKU and controls for overweight and obesity prevalence, BMI, waist circumference, and % body fat. Overweight prevalence was higher in patients with poor metabolic control and patients aged 10–16 years.	Moderate
Doulgeraki et al., 2014 [35]	Greece	Cross-sectional	80 (48 classical, 32 HPA)	Yes (NBS)	5.0–18.0	37:43	mean ± SD: PKU: 344 ± 178 HPA: 222 ± 51.6	57 matched for age and gender (mean age difference: 0.6 y)	BMI z-score Body fat (%) Weight z-score	Children with PKU had significantly higher BMI and weight when compared to healthy children. Fat mass increased significantly during puberty in PKU patients, especially in those with poor dietary adherence, and HPA.	High
Mazzola et al., 2016 [38]	Brazil	Cross-sectional	27 (13 classical, 14 mild)	Yes (11 early and 16 late diagnosed)	6.0–25.0	13:14	range at the time of the study: 102–1660	27 matched for age and gender (age NA)	BMI (kg/m^2^) Body fat (%) Overweight prevalence (%)	No significant differences for anthropometric measures between patients with PKU and controls. PKU severity, time of diagnosis, or metabolic control had no effect on any body composition outcome measures.	High
Evans et al., 2017 [25]	Australia	Longitudinal prospective (2 y)	37 (NA)	Yes (NBS)	0.6–18.0 ^2^	24:13	NA	21 matched for age and gender (mean age difference: 0.0 y)	BMI z-score Body fat (%) Weight z-score	No significant differences for BMI z-score and % body fat mass between patients treated with phe-restricted diet only, patients treated with BH4 + diet (*n* = 5), and controls.	High
Hermida-Ameijeiras et al., 2017 [37]	Spain	Cross-sectional	41 (22 classical,19 mild–moderate)	Yes (early and late diagnosed)	6.0–50.0	30:11	NA	41 matched for age and gender (mean age difference: -2.9 y)	BMI (kg/m^2^) Overweight prevalence (%)	No significant differences for BMI between patients with PKU and controls. Patients on BH4 therapy had lower BMI than those without BH4 therapy. Patients with lower Phe tolerance had higher body weight.	High
Couce et al., 2018 [30]	Spain	Cross-sectional	83 (37 classical, 20 mild-moderate 26 HPA)	Yes (70 early and 13 late diagnosed)	4.0–52.0	49:34	median: classical: 484 mild-moderate: 242 HPA: 296	68 matched for age and gender (age: NA)	Overweight prevalence (%)	Significantly higher % of overweight in patients with PKU than in patients with HPA and healthy controls, especially in those with good metabolic control.	Moderate
Evans et al., 2019 [31]	UK	Longitudinal Prospective (1.4–1.7 y; until 2 y of age)	20 (14 classical, 3 mild 3 moderate)	Yes (NBS)	0.2–0.6 ^2^	6:14	mean ± SD: 249 ± 81	20 (18 matched for birth order and mother’s educational level) (mean age difference: 0.0 y)	BMI z-score Overweight prevalence (%) Weight z-score	No significant differences between patients with PKU and controls for weight, head circumference, or BMI. Boys had lower mean BMI z-scores across both groups (PKU and controls).	Moderate
Azabdaftari et al., 2019 [36]	Germany	Cross-sectional	23 ^3^ (19 classical, 4 mild)	Yes (NBS)	18.0–47.0	10:13	mean ± SD: 1132 ± 321 (F: 1209 ± 316; M: 1068 ± 325)	28 healthy with similar age (mean age difference: -0.7 y)	BMI (kg/m^2^)	Patients with PKU had significantly higher BMI than controls. Patients with poor metabolic control also had significantly higher BMI.	High
Sailer et al., 2020 [18]	USA	Cross-sectional	30 (30 classical)	Yes (NBS)	5.0–16.0	12:18	mean ± SD: 392 ± 184	30 matched for age and gender (mean age difference: -0.1 y)	BMI (kg/m^2^) Body fat (%) Overweight prevalence (%)	No significant differences for BMI between patients with PKU and controls. Male subjects with PKU had significantly higher fat mass % and lower lean body mass % compared to male controls.	High

Abbreviations: BH4: sapropterin; BMI: body mass index; F: female; HPA: hyperphenylalaninaemia; M: male; NA: not available; NBS: newborn screening; Phe: phenylalanine; PKU: phenylketonuria; SD: standard deviation; UK: United Kingdom; USA: United States of America; y years. ^1^ Assessed using the National Institutes of Health (NIH) Quality Assessment Tool for Observational Cohort and Cross-Sectional Studies; ^2^ at baseline; ^3^ two patients refused physical examination.

## Supplementary Materials

The following are available online at https://www.mdpi.com/article/10.3390/nu13103443/s1, Figure S1: Risk of bias summary: Review authors’ judgements about each risk of bias item for each included study. Figure S2: Forest plot comparing the BMI between patients with PKU and healthy controls among studies with moderate and high risk of bias. Figure S3: Forest plot comparing the BMI between patients with PKU and healthy controls among studies including only early diagnosed patients and studies including both early and late diagnosed patients. Figure S4: Forest plot comparing the BMI between patients with PKU and healthy controls among studies including only children and adolescents; studies including only adults; and studies including children, adolescents, and adults. Figure S5: Forest plot comparing the BMI between patients with PKU and healthy controls among studies including both patients taking BH4 and patients not taking BH4, as well as studies including only patients not taking BH4. Figure S6: Forest plot comparing the BMI between patients with PKU and healthy controls among studies including patients with mixed phenotypes and studies including only patients with classical PKU. Figure S7: Publication bias plot. The SMD of BMI is plotted on the *x*-axis and the SE of the SMD is plotted on the *y*-axis. Table S1: Syntax of Mesh/Emtree terms per database. Table S2: Syntax of title, abstract, and author keyword per database. Table S3: Studies excluded from the systematic review with reasons. Table S4: Summary of between-group meta-analysis results. Table S5: NutriGrade assessment of the quality of the evidence.
