# Supplementary material for: Is the Phenylalanine-Restricted Diet a Risk Factor for Overweight or Obesity in Patients with Phenylketonuria (PKU)? A Systematic Review and Meta-Analysis
Source: Nutrients. 2021 Sep 28;13(10):3443. doi: 10.3390/nu13103443 (PMC8538431; doi:10.3390/nu13103443)
Supplement: Supplementary file 1 [file nutrients-13-03443-s001.zip › nutrients-1336975-supplementary.pdf]

Supplementary Materials

Section A – Search details

Databases:
Three databases were searched
1. Pubmed
2. Cochrane
3. Embase (Ovid)

A combination was used of:
- Mesh terms (Medical Subject Headings) from Pubmed and Cochrane or Emtree terms from Embase
- Text-word search in title, abstract and author keywords.

Entry terms:

1. "phenylketonurias"[Mesh]
2. "phenylalanine hydroxylase/deficiency"[Mesh]
3. hyperphenylalaninaemia OR hyperphenylalaninemia
4. phenylalanine hydroxylase deficiency OR PAH deficiency
5. phenylketonuri*
6. pku
7. 1 or 2 or 3 or 4 or 5 or 6
8. "Body Fat Distribution"[Mesh]
9. "Body Mass Index"[Mesh]
10. "Body Weight Changes"[Mesh]
11. "Diabetes Mellitus"[Mesh]
12. "Overweight"[Mesh]
13. "Glucose Intolerance"[Mesh]
14. adiposity
15. anthropometry or anthropometric
16. body composition
17. body fat
18. body mass index or bmi
19. body weight or body weights
20. diabetes
21. glucose intolerance or glucose tolerance
22. obesity
23. overweight
24. 8 or 9 or 10 or 11 or 11 or 12 or 13 or 14 or 15 or 16 or 17 or 18 or 19 or 20 or 21 or 22 or 23
25. 7 and 24

For Embase the following publications types were excluded: books or chapter or conference abstract or editorial or note or tombstone

Table S1 - Syntax of Mesh/Emtree terms per database

Entry number	Pubmed	Cochrane	Embase (OVID)
1	"phenylketonurias"[Mesh]	MeSH descriptor: [phenylketonurias] explode all trees	exp phenylketonuria/<br>exp hyperphenylalaninemia/
2	"phenylalanine hydroxylase/deficiency"[Mesh]	MeSH descriptor: Phenylalanine Hydroxylase] explode all trees and with qualifier(s): [deficiency – DF]	exp phenylalanine 4 monooxygenase/ and deficiency.ti.ab.kw
8	"Body Fat Distribution"[Mesh]	MeSH descriptor: [Body Fat Distribution] explode all trees	exp body fat/<br>anthropometric parameters/
9	"Body Mass Index"[Mesh]	MeSH descriptor: [Body Mass Index] explode all trees	exp body mass/
10	"Body Weight Changes"[Mesh]	MeSH descriptor: [Body Weight Changes] explode all trees	exp body weight change/
11	"Diabetes Mellitus"[Mesh]	MeSH descriptor: [Diabetes Mellitus] explode all trees	exp diabetes mellitus/
12	"Overweight"[Mesh]	MeSH descriptor: [Overweight] explode all trees	exp obesity/
13	"Glucose Intolerance"[Mesh]	MeSH descriptor: [Glucose Intolerance] explode all trees	exp glucose intolerance/

Abbreviations: Mesh: Medical Subject Headings.

Table S2 - Syntax of title, abstract and author keyword per database

Entry number	Pubmed	Cochrane	Embase (OVID)
3-6, 14-23	[tiab]	:ti,ab,kw	.ti,ab,kw.

Section B – List of excluded studies and reasons

Table S3 – Studies excluded from the systematic review with reasons

First author	Title	Year	Reason to exclude
Acosta [1]	Nutrient intakes and physical growth of children with phenylketonuria undergoing nutrition therapy	2003	Not relevant to PECO statement
Aldamiz-Echevarria [2]	Tetrahydrobiopterin therapy vs phenylalanine-restricted diet: impact on growth in PKU	2013	Not relevant to PECO statement
Aldamiz-Echevarria [3]	Anthropometric characteristics and nutrition in a cohort of PAH-deficient patients	2014	Not relevant to PECO statement
Aldamiz-Echevarria [4]	6R-tetrahydrobiopterin treated PKU patients below 4 years of age: Physical outcomes, nutrition and genotype	2015	Not relevant to PECO statement
Alfheeaid [5]	Impact of phenylketonuria type meal on appetite, thermic effect of feeding and postprandial fat oxidation	2018	Not relevant to PECO statement
Arnold [6]	Protein insufficiency and linear growth restriction in phenylketonuria	2002	Not relevant to PECO statement
Belanger-Quintana [7]	Physical development in patients with phenylketonuria on dietary treatment: A retrospective study	2011	Not relevant to PECO statement
Belanger-Quintana [8]	Multicentre study on long-term growth in patients with phenylketonuria	2014	Full text not available
Bushueva [9]	Evaluation of physical development in children with classical phenylketonuria	2015	Article in Russian
Buhrdel [10]	Effect of dietary measures on body weight and height of children with phenylketonuria in East Germany	1997	Article in German
Burrage [11]	High prevalence of overweight and obesity in females with phenylketonuria	2012	Not relevant to PECO statement
Burton [12]	Prevalence of comorbid conditions among adult patients diagnosed with phenylketonuria	2018	Not relevant to PECO statement
Caliman Camatta [13]	Body fat percentage in adolescents with phenylketonuria and associated factors	2020	Not relevant to PECO statement
Cobet [14]	Anthropometric measurements of children with phenylketonuria under diet therapy	1984	Article in German

Couce [15]	New insights in growth of phenylketonuric patients	2015	Not relevant to PECO statement
Couce [16]	Lipid profile status and other related factors in patients with Hyperphenylalaninemia	2016	Not relevant to PECO statement
Daly [17]	Glycomacropeptide: long-term use and impact on blood phenylalanine, growth and nutritional status in children with PKU	2019	Not relevant to PECO statement
Darba [18]	Characteristics, comorbidities, and use of healthcare resources of patients with phenylketonuria: a population-based study	2019	Not relevant to PECO statement
Dobbelaere [19]	Evaluation of nutritional status and pathophysiology of growth retardation in patients with phenylketonuria	2003	Not relevant to PECO statement
Gokmen Ozel [20]	Overweight and obesity in PKU: The results from 8 centres in Europe and Turkey	2014	Not relevant to PECO statement
Holtzman [21]	Termination of restricted diet in children with phenylketonuria: a randomized controlled study	1975	Not relevant to PECO statement
Jani [22]	Protein intake and physical activity are associated with body composition in individuals with phenylalanine hydroxylase deficiency	2017	Not relevant to PECO statement
Kanufre [23]	Metabolic syndrome in children and adolescents with phenylketonuria	2015	Not relevant to PECO statement
Lambruschini [24]	Clinical and nutritional evaluation of phenylketonuric patients on tetrahydrobiopterin monotherapy	2005	Not relevant to PECO statement
Lluch Fernandez [25]	Phenylketonuria. Treatment and developmental control	1988	Article in Spanish
Nara de Freitas de Almeida [26]	Nutritional and metabolic parameters of children and adolescents with phenylketonuria	2020	Not relevant to PECO statement
McBurnie [27]	Physical growth of children treated for phenylketonuria	1991	Not relevant to PECO statement
Moretti [28]	Dietary glycaemic index, glycaemic load and metabolic profile in children with phenylketonuria	2017	Not relevant to PECO statement
Oztruk [29]	Overweight and obesity in children under phenylalanine restricted diet	2018	Not relevant to PECO statement
Pinto [30]	Nutritional status in patients with phenylketonuria using glycomacropeptide as their major protein source	2017	Not relevant to PECO statement
Robertson [31]	Body mass index in adult patients with diet-treated phenylketonuria	2013	Not relevant to PECO statement
Rocha [32]	Early dietary treated patients with phenylketonuria can achieve normal growth and body composition	2013	Sample overlap

Sanlier [33]	Determination of anthropometric measurements and nutritional status of children with Phenylketonuria	2012	Not relevant to PECO statement
Scaglioni [34]	Body mass index rebound and overweight at 8 years of age in hyperphenylalaninemia children	2004	Not relevant to PECO statement
Stroup [35]	Sex differences in body composition and bone mineral density in phenylketonuria: A cross-sectional study	2018	Not relevant to PECO statement
Tansek [36]	Long-term BH4 (sapropterin) treatment of children with hyperphenylalaninemia - effect on median Phe/Tyr ratios	2016	Not relevant to PECO statement
Thiele [37]	Growth and final height among children with phenylketonuria	2017	Not relevant to PECO statement
Trefz [38]	Clinical burden of illness in patients with phenylketonuria (PKU) and associated comorbidities - a retrospective study of German health insurance claims data	2019	Not relevant to PECO statement
Walkowiak [39]	Overweight in classical phenylketonuria children: A retrospective cohort study	2019	Not relevant to PECO statement
White [40]	Excess weight among children with phenylketonuria	1982	Not relevant to PECO statement
Williams [41]	Plasma cholesterol in adults with phenylketonuria	2015	Not relevant to PECO statement

Abbreviations: PECO: Population, Exposure, Comparator, Outcome.

Section C – Supplemental Figures and Tables

	1. Research question	2. Study population	3. Participation rate	4. Recruitment criteria	5. Sample size justification	6. Exposure assessed prior to outcome measurement	7. Sufficient timeframe to see an effect	8. Different levels of exposure of interest	9. Exposure measures and assessment	10. Repeated exposure assessment	11. Outcome measures	12. Blinding of outcome assessors	13. Follow-up rate	14. Statistical analysis
Albersen 2010	+	−	+	+	+	−	+	?	−	?	+	−	?	−
Allen 1995	+	−	?	+	+	−	+	−	−	?	+	−	?	−
Allen 1996	+	−	?	+	−	+	+	?	+	−	+	−	−	−
Azabdaftari 2019	+	−	+	+	+	−	+	−	+	?	+	−	?	−
Couce 2018	+	+	?	+	−	−	+	+	+	?	+	−	?	−
Doulgeraki 2014	+	−	?	+	−	−	+	+	−	?	+	−	?	−
Evans 2017	+	−	?	+	−	+	+	+	+	+	+	−	?	−
Evans 2019	+	−	?	+	+	+	+	−	+	+	+	−	+	+
Fisberg 1999	+	−	?	−	−	−	+	−	+	?	+	−	?	−
Hermida-Ameijeiras 2017	+	+	?	+	−	−	+	−	−	?	+	−	?	−
Huemer 2007	+	−	?	+	−	+	+	?	+	+	+	−	+	−
Mazzola 2016	+	−	?	+	−	−	+	−	−	?	+	−	?	−
Rocha 2012	+	+	?	+	+	−	+	+	+	?	+	−	?	+
Sailer 2020	+	−	?	+	−	−	+	−	+	?	+	−	?	−
Schulpis 2000	+	−	?	−	−	−	+	+	+	?	+	−	?	−

Figure S1. Risk of bias summary: review authors' judgements about each risk of bias item for each included study. Green (+): low risk of bias; yellow (?): unclear risk of bias; red (-): high risk of bias.

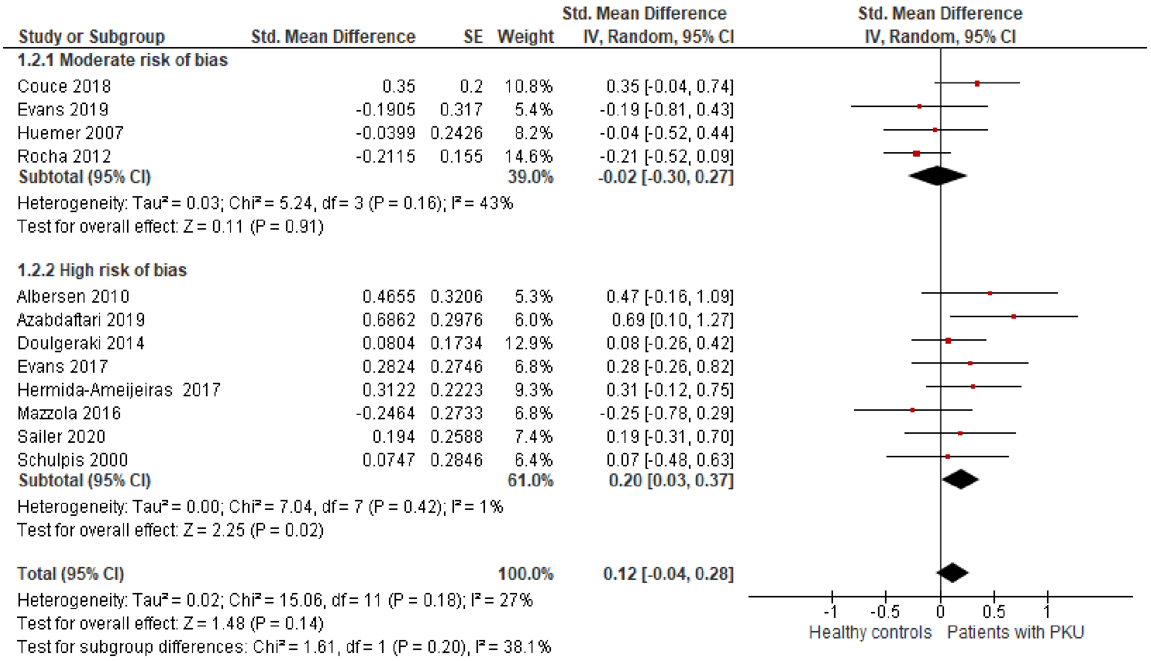

Figure S2. Forest plot comparing the BMI between patients with PKU and healthy controls among moderate and high risk of bias studies.

Abbreviations: BMI: Body Mass Index; CI: confidence interval; df: degrees of freedom; IV: inverse variance; PKU: phenylketonuria; SE: standard error; Std: standardized.

**Time of diagnosis:* Couce 2018 included 70 early and 13 late diagnosed patients, Hermida-Ameijeiras 2017 included both early and late diagnosed patients, Mazzola 2016 included 11 early and 16 late diagnosed patients, and Schulpis 2000 did not provide information on the time of diagnosis.

**Metabolic control:* Azabdaftari 2019 included only one patient with good metabolic control (Phe blood levels <600 μmol/L).

**BH4 treatment:* Couce 2018 included 10 (12%) patients taking BH4, Evans 2017 included 5 (14%), Hermida-Ameijeiras 2017 included 7 (17%), and Sailer 2020 included 4 (13%).

Study or Subgroup	Std. Mean Difference	SE	Weight	Std. Mean Difference IV, Random, 95% CI
**1.3.1 Only early diagnosed patients**				
Albersen 2010	0.4655	0.3206	5.9%	0.47 [-0.16, 1.09]
Azabdaftari 2019	0.6862	0.2976	6.6%	0.69 [0.10, 1.27]
Doulgeraki 2014	0.0804	0.1734	13.3%	0.08 [-0.26, 0.42]
Evans 2017	0.2824	0.2746	7.4%	0.28 [-0.26, 0.82]
Evans 2019	-0.1905	0.317	6.0%	-0.19 [-0.81, 0.43]
Huemer 2007	-0.0399	0.2426	8.9%	-0.04 [-0.52, 0.44]
Rocha 2012	-0.2115	0.155	14.9%	-0.21 [-0.52, 0.09]
Sailer 2020	0.194	0.2588	8.1%	0.19 [-0.31, 0.70]
**Subtotal (95% CI)**			**71.2%**	**0.11 [-0.10, 0.31]**
Heterogeneity: Tau² = 0.03; Chi² = 10.82, df = 7 (P = 0.15); I² = 35%				
Test for overall effect: Z = 1.00 (P = 0.32)				
**1.3.2 Early and late diagnosed patients**				
Couce 2018	0.35	0.2	11.4%	0.35 [-0.04, 0.74]
Hermida-Ameijeiras 2017	0.3122	0.2223	10.0%	0.31 [-0.12, 0.75]
Mazzola 2016	-0.2464	0.2733	7.5%	-0.25 [-0.78, 0.29]
**Subtotal (95% CI)**			**28.8%**	**0.18 [-0.17, 0.52]**
Heterogeneity: Tau² = 0.04; Chi² = 3.49, df = 2 (P = 0.18); I² = 43%				
Test for overall effect: Z = 1.01 (P = 0.31)				
**Total (95% CI)**			**100.0%**	**0.13 [-0.05, 0.30]**
Heterogeneity: Tau² = 0.03; Chi² = 15.05, df = 10 (P = 0.13); I² = 34%				
Test for overall effect: Z = 1.44 (P = 0.15)				
Test for subgroup differences: Chi² = 0.12, df = 1 (P = 0.73), I² = 0%				

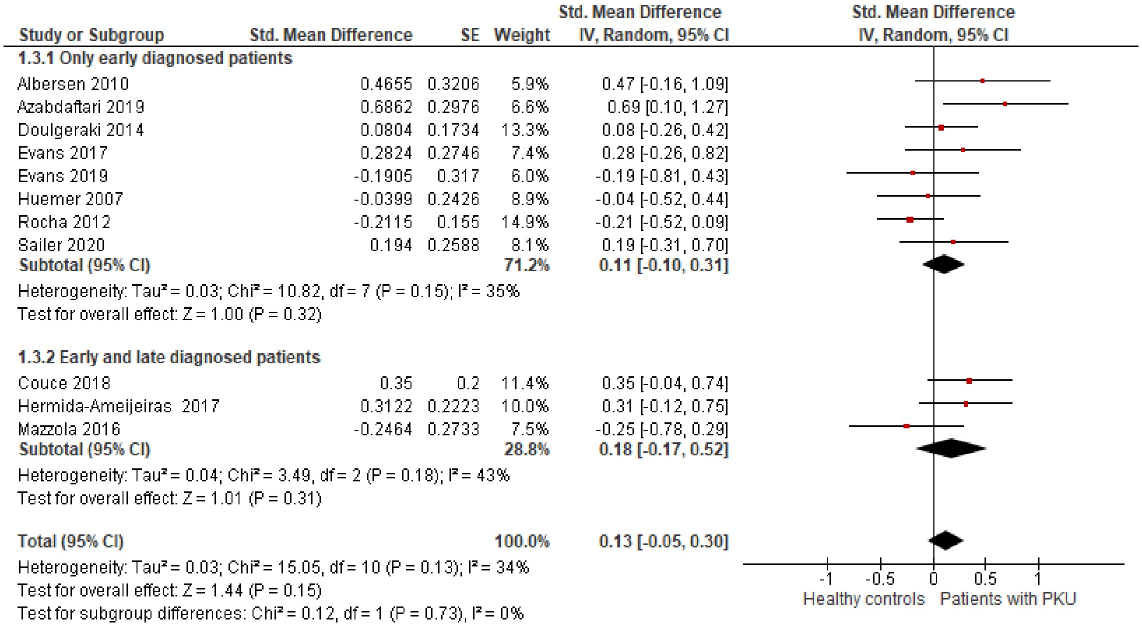

Figure S3. Forest plot comparing the BMI between patients with PKU and healthy controls among studies including only early diagnosed patients and studies including both early and late diagnosed patients. Abbreviations: BMI: Body Mass Index; CI: confidence interval; df: degrees of freedom; IV: inverse variance; PKU: phenylketonuria; SE: standard error; Std: standardized.

*<u>*Moderate risk of bias:*</u> Couce 2018, Evans 2019, Huemer 2007 and Rocha 2012.

*<u>*High risk of bias:*</u> Albersen 2010, Azabdaftari 2019, Doulgeraki 2014, Evans 2017, Hermida-Ameijeiras 2017, Mazzola 2016, Sailer 2020 and Schulpis 2000.

*<u>*Time of diagnosis:*</u> Couce 2018 included 70 early and 13 late diagnosed patients, Hermida-Ameijeiras 2017 included both early and late diagnosed patients, Mazzola 2016 included 11 early and 16 late diagnosed patients, and Schulpis 2000 did not provide information on the time of diagnosis.

*<u>*Metabolic control:*</u> Azabdaftari 2019 included only one patient with good metabolic control (Phe blood levels <600 μmol/L).

*<u>*BH4 treatment:*</u> Couce 2018 included 10 (12%) patients taking BH4, Evans 2017 included 5 (14%), Hermida-Ameijeiras 2017 included 7 (17%), and Sailer 2020 included 4 (13%).

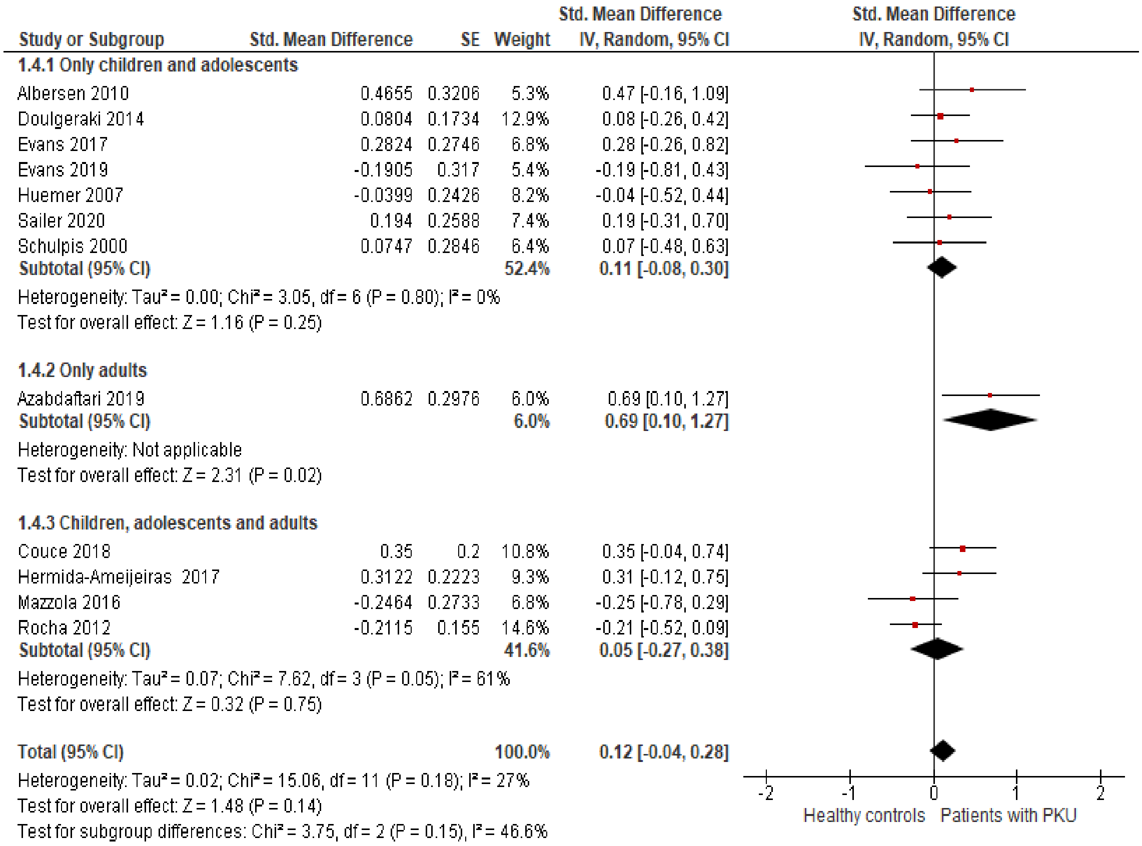

Figure S4. Forest plot comparing the BMI between patients with PKU and healthy controls among studies including only children and adolescents, studies including only adults, and studies including children, adolescents and adults.

Abbreviations: BMI: Body Mass Index; CI: confidence interval; df: degrees of freedom; IV: inverse variance; PKU: phenylketonuria; SE: standard error; Std: standardized.

**Moderate risk of bias:* Couce 2018, Evans 2019, Huemer 2007 and Rocha 2012.

**High risk of bias:* Albersen 2010, Azabdaftari 2019, Doulgeraki 2014, Evans 2017, Hermida-Ameijeiras 2017, Mazzola 2016, Sailer 2020 and Schulpis 2000.

**Time of diagnosis:* Couce 2018 included 70 early and 13 late diagnosed patients, Hermida-Ameijeiras 2017 included both early and late diagnosed patients, Mazzola 2016 included 11 early and 16 late diagnosed patients, and Schulpis 2000 did not provide information on the time of diagnosis.

**Metabolic control:* Azabdaftari 2019 included only one patient with good metabolic control (Phe blood levels <600 μmol/L).

**BH4 treatment:* Couce 2018 included 10 (12%) patients taking BH4, Evans 2017 included 5 (14%), Hermida-Ameijeiras 2017 included 7 (17%), and Sailer 2020 included 4 (13%).

Study or Subgroup	Std. Mean Difference	SE	Weight	Std. Mean Difference IV, Random, 95% CI
**1.5.1 Taking BH4 and not taking BH4**				
Couce 2018	0.35	0.2	10.8%	0.35 [-0.04, 0.74]
Evans 2017	0.2824	0.2746	6.8%	0.28 [-0.26, 0.82]
Hermida-Ameijeiras 2017	0.3122	0.2223	9.3%	0.31 [-0.12, 0.75]
Sailer 2020	0.194	0.2588	7.4%	0.19 [-0.31, 0.70]
**Subtotal (95% CI)**			**34.3%**	**0.30 [0.07, 0.52]**
Heterogeneity: Tau² = 0.00; Chi² = 0.24, df = 3 (P = 0.97); I² = 0%				
Test for overall effect: Z = 2.53 (P = 0.01)				
**1.5.2 Not taking BH4**				
Albersen 2010	0.4655	0.3206	5.3%	0.47 [-0.16, 1.09]
Azabdaftari 2019	0.6862	0.2976	6.0%	0.69 [0.10, 1.27]
Doulgeraki 2014	0.0804	0.1734	12.9%	0.08 [-0.26, 0.42]
Evans 2019	-0.1905	0.317	5.4%	-0.19 [-0.81, 0.43]
Huemer 2007	-0.0399	0.2426	8.2%	-0.04 [-0.52, 0.44]
Mazzola 2016	-0.2464	0.2733	6.8%	-0.25 [-0.78, 0.29]
Rocha 2012	-0.2115	0.155	14.6%	-0.21 [-0.52, 0.09]
Schulpis 2000	0.0747	0.2846	6.4%	0.07 [-0.48, 0.63]
**Subtotal (95% CI)**			**65.7%**	**0.04 [-0.17, 0.24]**
Heterogeneity: Tau² = 0.03; Chi² = 10.76, df = 7 (P = 0.15); I² = 35%				
Test for overall effect: Z = 0.33 (P = 0.74)				
**Total (95% CI)**			**100.0%**	**0.12 [-0.04, 0.28]**
Heterogeneity: Tau² = 0.02; Chi² = 15.06, df = 11 (P = 0.18); I² = 27%				
Test for overall effect: Z = 1.48 (P = 0.14)				
Test for subgroup differences: Chi² = 2.71, df = 1 (P = 0.10), I² = 63.1%				

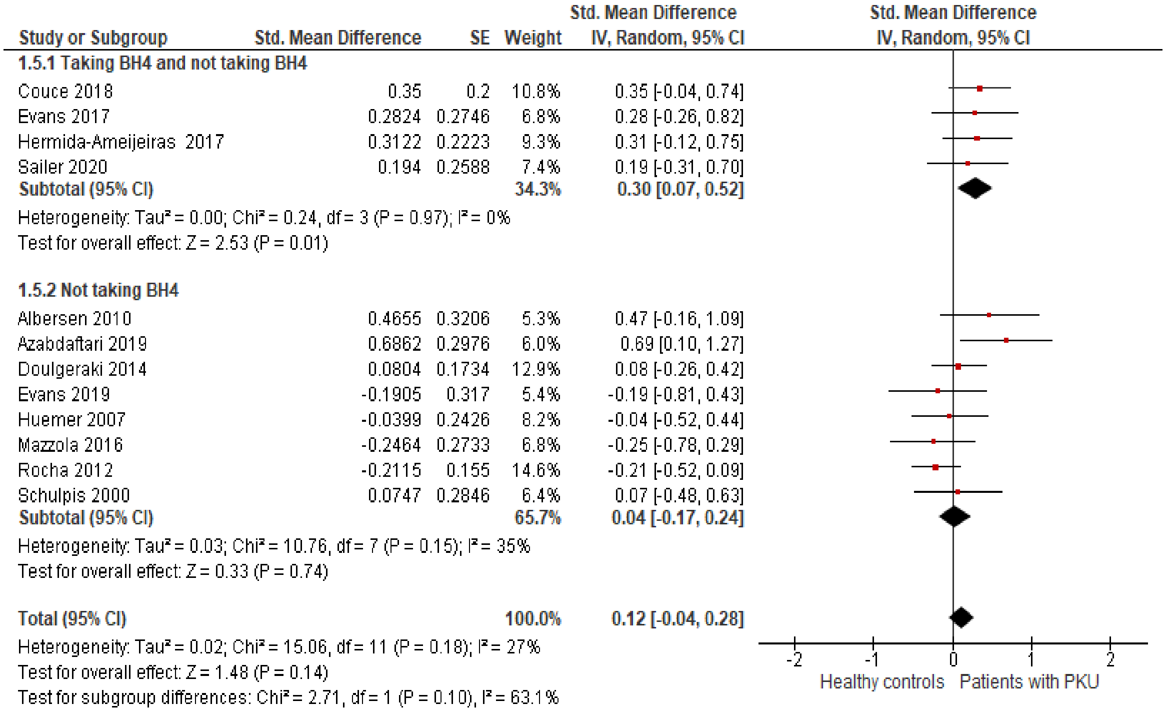

Figure S5. Forest plot comparing the BMI between patients with PKU and healthy controls among studies including both patients taking BH4 and patients not taking BH4, and studies including only patients not taking BH4.

Abbreviations: BH4: sapropterin; BMI: Body Mass Index; CI: confidence interval; df: degrees of freedom; IV: inverse variance; PKU: phenylketonuria; SE: standard error; Std: standardized.

<u>Moderate risk of bias:</u> Couce 2018, Evans 2019, Huemer 2007 and Rocha 2012.

<u>High risk of bias:</u> Albersen 2010, Azabdaftari 2019, Doulgeraki 2014, Evans 2017, Hermida-Ameijeiras 2017, Mazzola 2016, Sailer 2020 and Schulpis 2000.

<u>Time of diagnosis:</u> Couce 2018 included 70 early and 13 late diagnosed patients, Hermida-Ameijeiras 2017 included both early and late diagnosed patients, Mazzola 2016 included 11 early and 16 late diagnosed patients, and Schulpis 2000 did not provide information on the time of diagnosis.

<u>Metabolic control:</u> Azabdaftari 2019 included only one patient with good metabolic control (Phe blood levels <600 μmol/L).

<u>BH4 treatment:</u> Couce 2018 included 10 (12%) patients taking BH4, Evans 2017 included 5 (14%), Hermida-Ameijeiras 2017 included 7 (17%), and Sailer 2020 included 4 (13%).

Study or Subgroup	Std. Mean Difference	SE	Weight	Std. Mean Difference IV, Random, 95% CI
**1.6.1 Patients with mixed phenotypes**				
Azabdaftari 2019	0.6862	0.2976	6.6%	0.69 [0.10, 1.27]
Couce 2018	0.35	0.2	11.5%	0.35 [-0.04, 0.74]
Doulgeraki 2014	0.0804	0.1734	13.5%	0.08 [-0.26, 0.42]
Evans 2019	-0.1905	0.317	5.9%	-0.19 [-0.81, 0.43]
Hermida-Ameijeiras 2017	0.3122	0.2223	10.0%	0.31 [-0.12, 0.75]
Mazzola 2016	-0.2464	0.2733	7.5%	-0.25 [-0.78, 0.29]
Rocha 2012	-0.2115	0.155	15.2%	-0.21 [-0.52, 0.09]
**Subtotal (95% CI)**			**70.2%**	**0.10 [-0.14, 0.34]**
Heterogeneity: Tau² = 0.05; Chi² = 12.75, df = 6 (P = 0.05); I² = 53%				
Test for overall effect: Z = 0.82 (P = 0.41)				
**1.6.2 Only patients with classical PKU**				
Albersen 2010	0.4655	0.3206	5.8%	0.47 [-0.16, 1.09]
Huemer 2007	-0.0399	0.2426	8.9%	-0.04 [-0.52, 0.44]
Sailer 2020	0.194	0.2588	8.1%	0.19 [-0.31, 0.70]
Schulpis 2000	0.0747	0.2846	7.0%	0.07 [-0.48, 0.63]
**Subtotal (95% CI)**			**29.8%**	**0.14 [-0.12, 0.41]**
Heterogeneity: Tau² = 0.00; Chi² = 1.68, df = 3 (P = 0.64); I² = 0%				
Test for overall effect: Z = 1.04 (P = 0.30)				
**Total (95% CI)**			**100.0%**	**0.11 [-0.06, 0.28]**
Heterogeneity: Tau² = 0.02; Chi² = 14.61, df = 10 (P = 0.15); I² = 32%				
Test for overall effect: Z = 1.27 (P = 0.20)				
Test for subgroup differences: Chi² = 0.05, df = 1 (P = 0.82), I² = 0%				

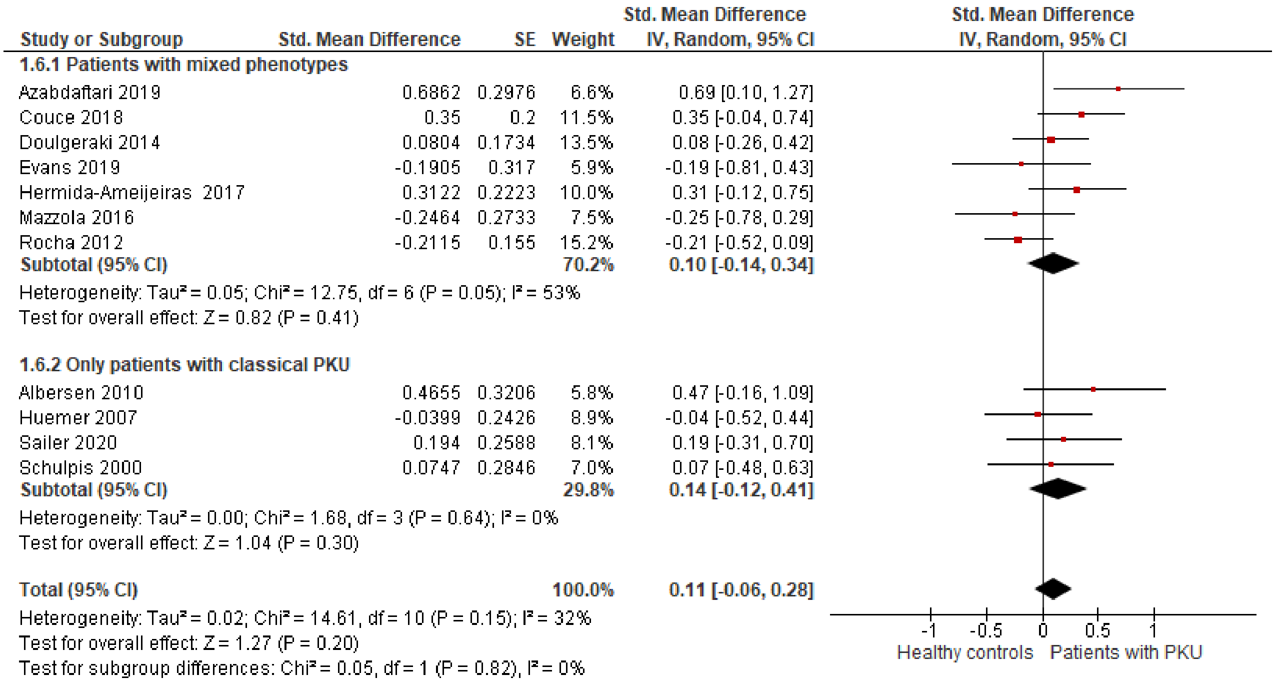

Figure S6. Forest plot comparing the BMI between patients with PKU and healthy controls among studies including patients with mixed phenotypes and studies including only patients with classical PKU.

Abbreviations: BMI: Body Mass Index; CI: confidence interval; df: degrees of freedom; IV: inverse variance; PKU: phenylketonuria; SE: standard error; Std: standardized.

*_Moderate risk of bias:_ Couce 2018, Evans 2019, Huemer 2007 and Rocha 2012.

*_High risk of bias:_ Albersen 2010, Azabdaftari 2019, Doulgeraki 2014, Evans 2017, Hermida-Ameijeiras 2017, Mazzola 2016, Sailer 2020 and Schulpis 2000.

*_Time of diagnosis:_ Couce 2018 included 70 early and 13 late diagnosed patients, Hermida-Ameijeiras 2017 included both early and late diagnosed patients, Mazzola 2016 included 11 early and 16 late diagnosed patients, and Schulpis 2000 did not provide information on the time of diagnosis.

*_Metabolic control:_ Azabdaftari 2019 included only one patient with good metabolic control (Phe blood levels <600 μmol/L).

*_BH4 treatment:_ Couce 2018 included 10 (12%) patients taking BH4, Evans 2017 included 5 (13%), Hermida-Ameijeiras 2017 included 7 (17%), and Sailer 2020 included 4 (13%).

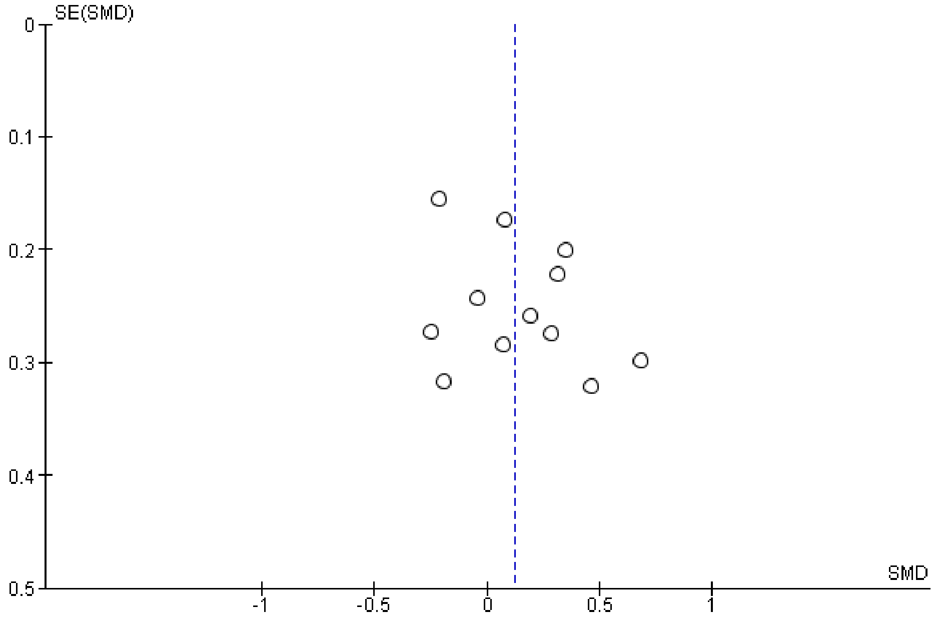

Figure S7. Publication bias plot. The SMD of BMI is plotted on the x axis and the SE of the SMD is plotted on the y axis. The vertical dotted line denotes the mean value of the SMDs reported by the 12 included studies.
Abbreviations: BMI: body mass index; SE: standard error; SMD: standardized mean difference.

Table S4. Summary of between-group meta-analysis results.

Outcome measure	Subgroup	Studies, n (references)	Cases, n		SMD (95% CI) [1]	*P* value	Heterogeneity test		Main conclusion
			PKU	controls			I^2 (%)	*P* value	
BMI	Phenotype (per participant)								
	Mild PKU	4 [42-45]	68	195	-	0.36	0	0.45	It is not possible to draw a conclusion from these results
	HPA	3 [43,45,46]	76	204	-	0.15	50	0.14	
BMI	Gender (per participant)								
	Males	6 [42,44-48]	123	110	-	0.52	39	0.14	It is not possible to draw a conclusion from these results
	Females	6 [42,44-48]	105	124	-	0.01	0	0.67	
BMI	Metabolic control (per participant) [2]								
	Poor metabolic control	5 [44-48]	41	206	-	0.08	58	0.05	It is not possible to draw a conclusion from these results
	Good metabolic control	5 [44-48]	192	206	-	0.31	2	0.39	
Body Fat %	Overall	8 [45-52]	348	326	-	0.71	60	0.02	It is not possible to draw a conclusion from these results
	Method								
	Air-displacement plethysmography	1 [47]	20	20	-	0.003	NA	NA	
	Skinfold-thickness	2 [49,50]	65	92	-	0.03	0	0.71	
	Dual X-ray absorptiometry	1 [46]	80	57	-	0.72	NA	NA	
	Bioelectrical impedance analysis	4 [45,48,51,52]	183	157	-	0.60	20	0.29	

Abbreviations: BMI: Body Mass Index; CI: confidence interval; HPA: hyperphenylalaninaemia; NA: not applicable; PKU: phenylketonuria; SMD: standardized mean difference. SMD and CI were not presented because the analysis excluded several studies or had a high heterogeneity. [1] Based on random-effects meta-analysis. [2] A cut-off of <360 and <600 µmol/L was used to define good metabolic control below and above 12 years of age, respectively.

Table S5. NutriGrade assessment of the quality of the evidence.

Outcome	Studies, n	Risk of Bias	Precision	Heterogeneity	Directness	Publication Bias	Funding Bias	Effect-size	Dose-response	Final score
BMI	12 [42-48,51-55]	1	1	1	1	0.5	0	0	0	4.5<br>low
Body fat %	8 [45-52]	0.5	0	0.4	1	0	0	0	0	1.9<br>very low

Abbreviations: BMI: Body Mass Index; PKU: phenylketonuria.

Section D – Guide to Assess the Quality of Observational Cohort and Cross-Sectional Studies

1. **Was the research question or objective in this paper clearly stated?**
   - Did the authors describe their goal in conducting this research? If so, the answer should be 'yes'.
   - If the authors did not describe their goal, the answer should be 'no'.

2. **Was the study population clearly specified and defined?**
   - Did the authors describe the group of people from which the study participants were selected or recruited, using demographics, location, and time period? If so, the answer should be 'yes'.
     Example: *Patients with PKU (who) followed at a PKU centre (where) between January 1, 2010 and December 31, 2012 (when).*
   - If the authors did not describe the group of people from which the study participants were selected or recruited, using demographics, location, and time period, the answer should be 'no'.

3. **Was the participation rate of eligible persons at least 50%?**
   - If the participation rate of eligible persons was at least 50%, the answer should be 'yes'.
   - If the participation rate of eligible persons was below 50%, the answer should be 'no'.
   - If the authors did not report this information, the answer should be 'not reported'.

4. **Were all the subjects selected or recruited from the same or similar populations (including the same time period)? Were inclusion and exclusion criteria for being in the study prespecified and applied uniformly to all participants?**
   - If all the subjects were selected or recruited from the same or similar populations (including the same time period), and inclusion and exclusion criteria for being in the study were prespecified and applied uniformly to all participants, the answer should be 'yes'.
   - If the subjects were selected or recruited from different populations, or inclusion and exclusion criteria for being in the study were not prespecified and applied uniformly to all participants, the answer should be 'no'.
   - If the authors did not report this information, the answer should be 'not reported'.

5. **Was a sample size justification, power description, or variance and effect estimates provided?**
   - If the authors present their reasons for selecting or recruiting the number of people included or analysed, the answer should be 'yes'.
   - If the authors note or discuss the statistical power of the study or give estimates of variance and/or estimates of effect size, the answer should be 'yes'.

- If the authors did not provide a sample size justification, power description, or variance and effect estimates, the answer should be ‘no’.

6. **For the analyses in this paper, were the exposure(s) of interest measured prior to the outcome(s) being measured?**
- In prospective cohort studies, if dietary intake was assessed before measuring the outcome, the answer to this question should be ‘yes’.
- In prospective cohort studies, if dietary intake was not assessed before measuring the outcome, the answer to this question should be ‘no’.
- In cross-sectional studies, the answer to this question should be ‘no’.

7. **Was the timeframe sufficient so that one could reasonably expect to see an association between exposure and outcome if it existed?**
- In cross-sectional and prospective cohort studies, if the patients began Phe-restriction in the neonatal period and maintained it throughout life, the answer to this question should be ‘yes’.
- If the patients did not start the Phe-restriction in the neonatal period or were unable to maintain it throughout life, the answer to this question should be ‘no’.

8. **For exposures that can vary in amount or level, did the study examine different levels of the exposure as related to the outcome (e.g., categories of exposure, or exposure measured as continuous variable)?**
- If the study compares different PKU phenotypes as related to the outcome (BMI or prevalence of overweight), the answer should be ‘yes’.
- If the study compares use of sapropterin treatment as related to the outcome (BMI or prevalence of overweight), the answer should be ‘yes’.
- If the study compares patients adhering strictly to the Phe-restricted diet with patients on a less restricted diet as related to the outcome (BMI or prevalence of overweight), the answer should be ‘yes’.
- If the study compares the time of diagnosis as related to the outcome (BMI or prevalence of overweight), the answer should be ‘yes’.
- If the study does not examine different levels of the exposure as related to the outcome (BMI or prevalence of overweight), the answer should be ‘no’.
- If the exposure does not vary in amount or level, the answer should be ‘not applicable’.

9. **Were the exposure measures (independent variables) clearly defined, valid, reliable, and implemented consistently across all study participants?**
- If the method used to measure the exposure was a three-day food record, a food diary, a 24-hour dietary recall or another validated dietary assessment tool, the answer should be ‘yes’.
- If no exposure measures were clearly defined, valid, reliable, and implemented consistently across all study participants, the answer should be ‘no’.

10. **Was the exposure(s) assessed more than once over time?**

- If the exposure to the Phe-restricted diet was measured more than once during the study period, the answer should be 'yes'.
- If the exposure to the Phe-restricted diet was only measured at the beginning of the study, the answer should be 'no'.
- In cross-sectional studies, in which exposure is only assessed once, the answer should be 'not applicable'.

11. Were the outcome measures (dependent variables) clearly defined, valid, reliable, and implemented consistently across all study participants?
- If BMI or prevalence of overweight were collected within the scope of the study, the answer should be 'yes'.
- If BMI or overweight prevalence were recorded at scheduled check-ups, the answer should be 'yes'.
- If BMI or overweight prevalence were collected from medical records, the answer should be 'yes'.
- If no outcome measures (BMI or prevalence of overweight) were clearly defined, valid, reliable, and implemented consistently across all study participants, the answer should be 'no'.

12. Were the outcome assessors blinded to the exposure status of participants?
- If the outcome assessors did not know whether participants were patients with PKU or healthy controls, the answer should be 'yes'.
- If the outcome assessors knew whether participants were patients with PKU or healthy controls, the answer should be 'no'.

13. Was loss to follow-up after baseline 20% or less?
- If loss to follow-up after baseline was 20% or less, the answer should be 'yes'.
- If loss to follow-up after baseline was more than 20%, the answer should be 'no'.
- In cross-sectional studies, the answer should be 'not applicable'.
- If the authors did not report this information, the answer should be 'not reported'.

14. Were key potential confounding variables (gender, age, educational level, occupation, race/ethnicity, living place, partnership status, household income, physical activity, dietary intake, puberty stage, among others) measured and adjusted statistically for their impact on the relationship between exposure(s) and outcome(s)?
- If at least two potential confounding variables were measured and adjusted for their impact on the relationship between exposure and outcome (BMI or prevalence of overweight), the answer should be 'yes'.
- If a logistic regression or other regression methods were used to account for the impact of at least two potential confounding variables on the relationship between exposure and outcome (BMI or prevalence of overweight), the answer should be 'yes'.

- If other statistical analyses were performed to control the impact on the relationship between exposure and outcome (BMI or prevalence of overweight) of at least two potential confounders, for instance stratification, the answer should be 'yes'.
- If no statistical adjustment was performed or a statistical analysis was performed to control the impact on the relationship between exposure and outcome (BMI or prevalence of overweight) of one potential confounder, the answer should be 'no'.

Rating of the overall Risk of Bias:

A. Non-fatal flaws – articles can still be classified as 'good':

- If the authors did not report anything about sample size, it just indicates they did not pay attention to whether the sample was sized enough to answer the research question (question 5).
- In cross-sectional studies, if the exposure was not assessed more than once over time (question 10).
- If outcome assessors were aware of participants' exposure status, since the outcome is objective (question 12).

B. **Moderate flaws** – articles with <u>four or more</u> of these flaws should be classified as 'poor'; articles with <u>three</u> of these flaws should be classified as 'fair'; articles with <u>two</u> <u>or less</u> of these flaws should be classified as 'good':

- If the authors did not describe the group of people from which the study participants were selected or recruited, using demographics, location, and time period (question 2).
- If the authors did not report the participation rate (question 3).
- If the time frame was not sufficient to see an effect (question 7).
- In prospective studies, if the exposure was not assessed more than once over time (question 10).
- If loss to follow-up after baseline was not reported or was 20% or more, since the acceptable 80% follow-up rate is just a general guideline (question 13).

C. **Fatal-flaws** – articles with <u>three or more</u> of these flaws should be classified as 'poor'; articles with <u>two or one</u> of these flaws should be classified as 'fair':

- If the authors did not clearly state the research question or objective (question 1).
- If less than 50% of eligible persons participated in the study, the study population may not adequately represent the target population (question 3).
- If patients were recruited from different populations or the inclusion and exclusion criteria were not used for all the subjects involved (question 4).
- If the exposure was not assessed prior to outcome measurement (question 6).
- If exposure can vary in amount or level, and the study did not examine different levels of the exposure as related to the outcome (question 8).
- If the methods used to measure exposure were not accurate and reliable or the exposure was not measured (question 9).
- If the methods used to measure outcomes were not accurate and reliable or the outcomes were not measured (question 11).
- If the authors did not control for potential confounders (question 14).

References

1. Acosta, P.B.; Yannicelli, S.; Singh, R.; Mofidi, S.; Steiner, R.; DeVincentis, E.; Jurecki, E.; Bernstein, L.; Gleason, S.; Chetty, M., et al. Nutrient intakes and physical growth of children with phenylketonuria undergoing nutrition therapy. *J. Am. Diet. Assoc.* **2003**, *103*, 1167–1173, doi:10.1016/s0002-8223(03)00983-0.
2. Aldámiz-Echevarría, L.; Bueno, M.A.; Couce, M.L.; Lage, S.; Dalmau, J.; Vitoria, I.; Andrade, F.; Llarena, M.; Blasco, J.; Alcalde, C., et al. Tetrahydrobiopterin therapy vs phenylalanine-restricted diet: impact on growth in PKU. *Mol. Genet. Metab.* **2013**, *109*, 331–338, doi:10.1016/j.ymgme.2013.05.017.
3. Aldámiz-Echevarría, L.; Bueno, M.A.; Couce, M.L.; Lage, S.; Dalmau, J.; Vitoria, I.; Andrade, F.; Blasco, J.; Alcalde, C.; Gil, D., et al. Anthropometric characteristics and nutrition in a cohort of PAH-deficient patients. *Clin. Nutr.* **2014**, *33*, 702–717, doi:10.1016/j.clnu.2013.09.011.
4. Aldámiz-Echevarría, L.; Bueno, M.A.; Couce, M.L.; Lage, S.; Dalmau, J.; Vitoria, I.; Llarena, M.; Andrade, F.; Blasco, J.; Alcalde, C., et al. 6R-tetrahydrobiopterin treated PKU patients below 4 years of age: Physical outcomes, nutrition and genotype. *Mol. Genet. Metab.* **2015**, *115*, 10–16, doi:10.1016/j.ymgme.2015.03.007.
5. Alfheeaid, H.; Gerasimidis, K.; Năstase, A.M.; Elhauge, M.; Cochrane, B.; Malkova, D. Impact of phenylketonuria type meal on appetite, thermic effect of feeding and postprandial fat oxidation. *Clin. Nutr.* **2018**, *37*, 851-857, doi:10.1016/j.clnu.2017.03.005.
6. Arnold, G.L.; Vladutiu, C.J.; Kirby, R.S.; Blakely, E.M.; Deluca, J.M. Protein insufficiency and linear growth restriction in phenylketonuria. *J. Pediatr.* **2002**, *141*, 243–246, doi:10.1067/mpd.2002.126455.
7. Belanger-Quintana, A.; Martínez-Pardo, M. Physical development in patients with phenylketonuria on dietary treatment: a retrospective study. *Mol. Genet. Metab.* **2011**, *104*, 480–484, doi:10.1016/j.ymgme.2011.08.002.
8. Bélanger-Quintana, A.; Stanescu, S.; Ahring, K.; Dokoupil, K.; Ozel, H.; Lammardo, A.; Macdonald, A.; Robert, M.; Rocha, J.; Rijn, M. Multicentre study on growth in PKU patients: preliminary results. In Proceedings of Annual Meeting of the Society-for-Inherited-Metabolic-Disorders (SIMD), California, USA; p. 289.
9. Bushueva, T.V.; Borovik, T.E.; Ladodo, K.S.; Kuzenkova, L.M.; Maslova, O.I.; Gevorkyan, A.K. Evaluation of physical development in children with classical phenylketonuria. *Vopr. Pitan.* **2015**, *84*, 34–43.
10. Bührdel, P.; Däbritz, S.; Theile, H. Effect of dietary measures on body weight and height of children with phenylketonuria in East Germany. *Klin. Padiatr.* **1997**, *209*, 26–29, doi:10.1055/s-2008-1043923.
11. Burrage, L.C.; McConnell, J.; Haesler, R.; O'Riordan, M.A.; Sutton, V.R.; Kerr, D.S.; McCandless, S.E. High prevalence of overweight and obesity in females with phenylketonuria. *Mol. Genet. Metab.* **2012**, *107*, 43–48, doi:10.1016/j.ymgme.2012.07.006.
12. Burton, B.K.; Jones, K.B.; Cederbaum, S.; Rohr, F.; Waisbren, S.; Irwin, D.E.; Kim, G.; Lilienstein, J.; Alvarez, I.; Jurecki, E., et al. Prevalence of comorbid conditions among adult patients diagnosed with phenylketonuria. *Mol. Genet. Metab.* **2018**, *125*, 228–234, doi:10.1016/j.ymgme.2018.09.006.
13. Camatta, G.C.; Kanufre, V.C.; Alves, M.R.A.; Soares, R.D.L.; Norton, R.C.; de Aguiar, M.J.B.; Starling, A.L.P. Body fat percentage in adolescents with phenylketonuria and associated factors. *Mol. Genet. Metab. Rep.* **2020**, *23*, 100595, doi:10.1016/j.ymgmr.2020.100595.
14. Cobet, G.; Grimm, H.; Seidlitz, G.; Theile, H. Anthropometric measurements of children with phenylketonuria under diet therapy. *Arztl. Jugendkd.* **1984**, *75*, 20–28.
15. Couce, M.L.; Guler, I.; Anca-Couce, A.; Lojo, M.; Mirás, A.; Leis, R.; Pérez-Muñuzuri, A.; Fraga, J.M.; Gude, F. New insights in growth of phenylketonuric patients. *Eur. J. Pediatr.* **2015**, *174*, 651–659, doi:10.1007/s00431-014-2446-8.
16. Couce, M.L.; Vitoria, I.; Aldámiz-Echevarría, L.; Fernández-Marmiesse, A.; Roca, I.; Llarena, M.; Sánchez-Pintos, P.; Leis, R.; Hermida, A. Lipid profile status and other related factors in patients with Hyperphenylalaninaemia. *Orphanet J. Rare Dis.* **2016**, *11*, 123, doi:10.1186/s13023-016-0508-x.
17. Daly, A.; Evans, S.; Chahal, S.; Santra, S.; Pinto, A.; Jackson, R.; Gingell, C.; Rocha, J.; Van Spronsen, F.J.; MacDonald, A. Glycomacropeptide: long-term use and impact on blood phenylalanine, growth and nutritional status in children with PKU. *Orphanet J. Rare Dis.* **2019**, *14*, 44, doi:10.1186/s13023-019-1011-y.
18. Darbà, J. Characteristics, comorbidities, and use of healthcare resources of patients with phenylketonuria: a population-based study. *J. Med. Econ.* **2019**, *22*, 1025–1029, doi:10.1080/13696998.2019.1636381.
19. Dobbelaere, D.; Michaud, L.; Debrabander, A.; Vanderbecken, S.; Gottrand, F.; Turck, D.; Farriaux, J.P. Evaluation of nutritional status and pathophysiology of growth retardation in patients with phenylketonuria. *J. Inherit. Metab. Dis.* **2003**, *26*, 1–11, doi:10.1023/a:1024063726046.

20. Gokmen Ozel, H.; Ahring, K.; Bélanger-Quintana, A.; Dokoupil, K.; Lammardo, A.M.; Robert, M.; Rocha, J.C.; Almeida, M.F.; van Rijn, M.; MacDonald, A. Overweight and obesity in PKU: The results from 8 centres in Europe and Turkey. *Mol. Genet. Metab. Rep.* **2014**, *1*, 483–486, doi:10.1016/j.ymgmr.2014.11.003.
21. Holtzman, N.A.; Welcher, D.W.; Mellits, E.D. Termination of restricted diet in children with phenylketonuria: a randomized controlled study. *N. Engl. J. Med.* **1975**, *293*, 1121–1124, doi:10.1056/nejm197511272932204.
22. Jani, R.; Coakley, K.; Douglas, T.; Singh, R. Protein intake and physical activity are associated with body composition in individuals with phenylalanine hydroxylase deficiency. *Mol. Genet. Metab.* **2017**, *121*, 104–110, doi:10.1016/j.ymgme.2017.04.012.
23. Kanufre, V.C.; Soares, R.D.; Alves, M.R.; Aguiar, M.J.; Starling, A.L.; Norton, R.C. Metabolic syndrome in children and adolescents with phenylketonuria. *J. Pediatr. (Rio J)* **2015**, *91*, 98–103, doi:10.1016/j.jped.2014.06.006.
24. Lambruschini, N.; Pérez-Dueñas, B.; Vilaseca, M.A.; Mas, A.; Artuch, R.; Gassió, R.; Gómez, L.; Gutiérrez, A.; Campistol, J. Clinical and nutritional evaluation of phenylketonuric patients on tetrahydrobiopterin monotherapy. *Mol. Genet. Metab.* 2005, 86 Suppl 1, S54-60, doi:10.1016/j.ymgme.2005.05.014.
25. Lluch Fernández, M.D.; Ramos Sánchez, I.; Marchante Cobos, C.; Pérez Pérez, G.; Martínez Martínez, J.J.; Estefanía Gallardo, C. [Phenylketonuria. Treatment and developmental control]. *An. Esp. Pediatr.* **1988**, *28*, 327–330.
26. de Almeida, B.N.F.; Laufer, J.A.; Mezzomo, T.R.; Shimada, N.C.; Furtado, I.H.F.; Dias, M.; Pereira, R.M. Nutritional and metabolic parameters of children and adolescents with phenylketonuria. *Clin Nutr ESPEN* **2020**, *37*, 44–49, doi:10.1016/j.clnesp.2020.03.024.
27. McBurnie, M.A.; Kronmal, R.A.; Schuett, V.E.; Koch, R.; Azeng, C.G. Physical growth of children treated for phenylketonuria. *Ann. Hum. Biol.* **1991**, *18*, 357–368, doi:10.1080/03014469100001662.
28. Moretti, F.; Pellegrini, N.; Salvatici, E.; Rovelli, V.; Banderali, G.; Radaelli, G.; Scazzina, F.; Giovannini, M.; Verduci, E. Dietary glycemic index, glycemic load and metabolic profile in children with phenylketonuria. *Nutr. Metab. Cardiovasc.* Dis. **2017**, *27*, 176–182, doi:10.1016/j.numecd.2016.11.002.
29. Ozturk, Y.; Gencpinar, P.; Erdur, B.; Tokgöz, Y.; Isik, I.; Akin, S.B. Overweight and obesity in children under phenylalanine restricted diet. *Hong Kong J. Pediatr.* **2018**, *23*, 169–172.
30. Pinto, A.; Almeida, M.F.; Ramos, P.C.; Rocha, S.; Guimas, A.; Ribeiro, R.; Martins, E.; Bandeira, A.; MacDonald, A.; Rocha, J.C. Nutritional status in patients with phenylketonuria using glycomacropeptide as their major protein source. *Eur. J. Clin. Nutr.* **2017**, *71*, 1230–1234, doi:10.1038/ejcn.2017.38.
31. Robertson, L.V.; McStravick, N.; Ripley, S.; Weetch, E.; Donald, S.; Adam, S.; Micciche, A.; Boocock, S.; MacDonald, A. Body mass index in adult patients with diet-treated phenylketonuria. *J. Hum. Nutr. Diet.* 2013, 26 Suppl 1, 1-6, doi:10.1111/jhn.12054.
32. Rocha, J.C.; van Spronsen, F.J.; Almeida, M.F.; Ramos, E.; Guimarães, J.T.; Borges, N. Early dietary treated patients with phenylketonuria can achieve normal growth and body composition. *Mol. Genet. Metab.* 2013, 110 Suppl, S40-43, doi:10.1016/j.ymgme.2013.10.009.
33. Sanlier, N.; Bakirel, A.N.; yassıbaş, E.; Uyar, B.; Sahin, G. Determination of anthropometric measurements and nutritional status of children with Phenylketonuria. *HealthMED* **2012**, *6*, 632–639.
34. Scaglioni, S.; Verduci, E.; Fiori, L.; Lammardo, A.M.; Rossi, S.; Radaelli, G.; Riva, E.; Giovannini, M. Body mass index rebound and overweight at 8 years of age in hyperphenylalaninaemic children. *Acta Paediatr.* **2004**, *93*, 1596–1600.
35. Stroup, B.M.; Hansen, K.E.; Krueger, D.; Binkley, N.; Ney, D.M. Sex differences in body composition and bone mineral density in phenylketonuria: A cross-sectional study. *Mol. Genet. Metab. Rep.* **2018**, *15*, 30–35, doi:10.1016/j.ymgmr.2018.01.004.
36. Tansek, M.Z.; Groselj, U.; Kelvisar, M.; Kobe, H.; Lampret, B.R.; Battelino, T. Long-term BH4 (sapropterin) treatment of children with hyperphenylalaninemia - effect on median Phe/Tyr ratios. *J. Pediatr. Endocrinol. Metab.* **2016**, *29*, 561–566, doi:10.1515/jpem-2015-0337.
37. Thiele, A.G.; Gausche, R.; Lindenberg, C.; Beger, C.; Arelin, M.; Rohde, C.; Mütze, U.; Weigel, J.F.; Mohnike, K.; Baerwald, C., et al. Growth and Final Height Among Children With Phenylketonuria. *Pediatrics* **2017**, *140*, doi:10.1542/peds.2017-0015.
38. Trefz, K.F.; Muntau, A.C.; Kohlscheen, K.M.; Altevers, J.; Jacob, C.; Braun, S.; Greiner, W.; Jha, A.; Jain, M.; Alvarez, I., et al. Clinical burden of illness in patients with phenylketonuria (PKU) and associated comorbidities - a retrospective study of German health insurance claims data. *Orphanet J. Rare Dis.* **2019**, *14*, 181, doi:10.1186/s13023-019-1153-y.

39. Walkowiak, D.; Kaluzny, L.; Bukowska-Posadzy, A.; Oltarzewski, M.; Staszewski, R.; Moczko, J.A.; Musielak, M.; Walkowiak, J. Overweight in classical phenylketonuria children: A retrospective cohort study. *Adv. Med. Sci.* **2019**, *64*, 409–414, doi:10.1016/j.advms.2019.08.001.
40. White, J.E.; Kronmal, R.A.; Acosta, P.B. Excess weight among children with phenylketonuria. *J. Am. Coll. Nutr.* **1982**, *1*, 293–303, doi:10.1080/07315724.1982.10718998.
41. Williams, R.A.; Hooper, A.J.; Bell, D.A.; Mamotte, C.D.; Burnett, J.R. Plasma cholesterol in adults with phenylketonuria. *Pathology* **2015**, *47*, 134–137, doi:10.1097/pat.0000000000000210.
42. Azabdaftari, A.; van der Giet, M.; Schuchardt, M.; Hennermann, J.B.; Plöckinger, U.; Querfeld, U. The cardiovascular phenotype of adult patients with phenylketonuria. *Orphanet J. Rare Dis.* **2019**, *14*, 213, doi:10.1186/s13023-019-1188-0.
43. Couce, M.L.; Sánchez-Pintos, P.; Vitoria, I.; De Castro, M.J.; Aldámiz-Echevarría, L.; Correcher, P.; Fernández-Marmiesse, A.; Roca, I.; Hermida, A.; Martínez-Olmos, M., et al. Carbohydrate status in patients with phenylketonuria. *Orphanet J. Rare Dis*. **2018**, *13*, 103, doi:10.1186/s13023-018-0847-x.
44. Evans, S.; Daly, A.; Wildgoose, J.; Cochrane, B.; Chahal, S.; Ashmore, C.; Loveridge, N.; MacDonald, A. Growth, Protein and Energy Intake in Children with PKU Taking a Weaning Protein Substitute in the First Two Years of Life: A Case-Control Study. *Nutrients* 2019, 11, doi:10.3390/nu11030552.
45. Rocha, J.C.; van Spronsen, F.J.; Almeida, M.F.; Soares, G.; Quelhas, D.; Ramos, E.; Guimarães, J.T.; Borges, N. Dietary treatment in phenylketonuria does not lead to increased risk of obesity or metabolic syndrome. *Mol. Genet. Metab.* **2012**, *107*, 659–663, doi:10.1016/j.ymgme.2012.10.006.
46. Doulgeraki, A.; Skarpalezou, A.; Theodosiadou, A.; Monopolis, I.; Schulpis, K. Body composition profile of young patients with phenylketonuria and mild hyperphenylalaninemia. *Int. J. Endocrinol. Metab.* 2014, 12, e16061, doi:10.5812/ijem.16061.
47. Albersen, M.; Bonthuis, M.; de Roos, N.M.; van den Hurk, D.A.; Carbasius Weber, E.; Hendriks, M.M.; de Sain-van der Velden, M.G.; de Koning, T.J.; Visser, G. Whole body composition analysis by the BodPod air-displacement plethysmography method in children with phenylketonuria shows a higher body fat percentage. *J. Inherit. Metab. Dis.* 2010, 33 Suppl 3, S283-288, doi:10.1007/s10545-010-9149-8.
48. Sailer, M.; Elizondo, G.; Martin, J.; Harding, C.O.; Gillingham, M.B. Nutrient intake, body composition, and blood phenylalanine control in children with phenylketonuria compared to healthy controls. *Mol. Genet. Metab. Rep.* **2020**, *23*, 100599–100599, doi:10.1016/j.ymgmr.2020.100599.
49. Allen, J.R.; Baur, L.A.; Waters, D.L.; Humphries, I.R.; Allen, B.J.; Roberts, D.C.; Gaskin, K.J. Body protein in prepubertal children with phenylketonuria. *Eur. J. Clin. Nutr.* **1996**, *50*, 178–186.
50. Allen, J.R.; McCauley, J.C.; Waters, D.L.; O'Connor, J.; Roberts, D.C.; Gaskin, K.J. Resting energy expenditure in children with phenylketonuria. *Am. J. Clin. Nutr.* **1995**, *62*, 797–801, doi:10.1093/ajcn/62.4.797.
51. Mazzola, P.N.; Nalin, T.; Castro, K.; van Rijn, M.; Derks, T.G.; Perry, I.D.; Mainieri, A.S.; Schwartz, I.V. Analysis of body composition and nutritional status in Brazilian phenylketonuria patients. *Mol. Genet. Metab. Rep.* **2016**, *6*, 16–20, doi:10.1016/j.ymgmr.2015.12.003.
52. Evans, M.; Truby, H.; Boneh, A. The relationship between dietary intake, growth and body composition in Phenylketonuria. *Mol. Genet. Metab.* **2017**, *122*, 36–42, doi:10.1016/j.ymgme.2017.07.007.
53. Hermida-Ameijeiras, A.; Crujeiras, V.; Roca, I.; Calvo, C.; Leis, R.; Couce, M.L. Arterial stiffness assessment in patients with phenylketonuria. *Medicine (Baltimore)* 2017, 96, e9322, doi:10.1097/md.0000000000009322.
54. Schulpis, K.H.; Papakonstantinou, E.D.; Tzamouranis, J. Plasma leptin concentrations in phenylketonuric patients. *Horm. Res.* **2000**, *53*, 32–35, doi:10.1159/000023510.
55. Huemer, M.; Huemer, C.; Möslinger, D.; Huter, D.; Stöckler-Ipsiroglu, S. Growth and body composition in children with classical phenylketonuria: results in 34 patients and review of the literature. *J. Inherit. Metab. Dis.* **2007**, *30*, 694–699, doi:10.1007/s10545-007-0549-3.
